# Supplementary material for: Climate-trait relationships exhibit strong habitat specificity in plant communities across Europe
Source: Nat Commun. 2023 Feb 9;14:712. doi: 10.1038/s41467-023-36240-6 (PMC9911725; doi:10.1038/s41467-023-36240-6)
Supplement: Supplementary file 1 — Supplementary Information [file 41467_2023_36240_MOESM1_ESM.docx]

**Supplementary Information**

Climate-trait relationships exhibit strong habitat specificity in plant communities across Europe

Stephan Kambach, Francesco Maria Sabatini, Fabio Attorre, Idoia Biurrun, Gerhard Boenisch, Gianmaria Bonari, Andraž Čarni, Maria Laura Carranza, Alessandro Chiarucci, Milan Chytrý, Jürgen Dengler, Emmanuel Garbolino, Valentin Golub, Behlül Güler, Ute Jandt, Jan Jansen, Anni Jašková, Borja Jiménez-Alfaro, Dirk Nikolaus Karger, Jens Kattge, Ilona Knollová, Gabriele Midolo, Jesper Erenskjold Moeslund, Remigiusz Pielech, Valerijus Rašomavičius, Solvita Rūsiņa, Jozef Šibík, Zvjezdana Stančić, Angela Stanisci, Jens-Christian Svenning, Sergey Yamalov_,_ Niklaus E. Zimmermann, Helge Bruelheide

**Supplementary Table S1 | Significance of random-slope interactions between climatic PCs and the broad, intermediate, and most narrowly defined habitats.** The table shows the estimated degrees of freedom (edf), the F-value, the significance of the random slope term (per habitat) and the conditional R² value from generalized additive mixed-effects models with the community-weighted trait mean as the dependent variable and the first four principal components of the 19 bioclimatic variables as fixed effects. Separate models were calculated for each combination of the four traits and the different levels of the habitat classification hierarchy.

| **Trait** | **Principal**  **component** | **Broad habitats**  (model 3) | | | **Intermediate habitats**  (model 4) | | | **Narrow habitats**  (model 5) | | |
| --- | --- | --- | --- | --- | --- | --- | --- | --- | --- | --- |
|  |  |  |  |  |  |  |  |  |  |  |
|  |  | **edf** | **F** |  | **edf** | **F** |  | **edf** | **F** |  |
| **Plant height** | PC1 | 6.98 | 18,775.03 | *** | 32.15 | 139,179.21 | *** | 148.82 | 103,096.79 | *** |
|  | PC2 | 6.91 | 10,830.14 | *** | 32.69 | 62,046.88 | *** | 146.62 | 219,973.43 | *** |
|  | PC3 | 6.96 | 8,445.17 | *** | 33.16 | 16,277.17 | *** | 160.44 | 56,098.94 | *** |
|  | PC4 | 6.9 | 2,810.69 | *** | 30.89 | 13,443.71 | *** | 144.37 | 73,512.46 | *** |
|  |  | R² = 0.81 | | | R² = 0.79 | | | R² = 0.92 | | |
|  |  |  |  |  |  |  |  |  |  |  |
| **Specific** | PC1 | 6.83 | 1,451.92 | *** | 30.25 | 7,710.95 | *** | 144.66 | 18,991.84 | *** |
| **Leaf area** | PC2 | 6.93 | 3,459.89 | *** | 34.2 | 23,190.89 | *** | 165.28 | 18,623.41 | *** |
|  | PC3 | 6.81 | 1,522.73 | *** | 27.96 | 2,271.03 | *** | 157.37 | 5,207.52 | *** |
|  | PC4 | 6.66 | 1,012.3 | *** | 33.68 | 2,796.43 | *** | 167.98 | 3,202.14 | *** |
|  |  | R² = 0.35 | | | R² = 0.51 | | | R² = 0.64 | | |
|  |  |  |  |  |  |  |  |  |  |  |
| **Seed mass** | PC1 | 6.94 | 4,682.41 | *** | 28.76 | 12,224.76 | *** | 144.41 | 10,902.1 | *** |
|  | PC2 | 6.85 | 3,169.56 | *** | 31.01 | 22,903.01 | *** | 134.1 | 11,459.02 | *** |
|  | PC3 | 6.95 | 3,181.44 | *** | 30.69 | 1,503.43 | *** | 149.72 | 4,394.84 | *** |
|  | PC4 | 6.62 | 287.96 | *** | 28.22 | 915.04 | *** | 128.93 | 2,134.4 | *** |
|  |  | R² = 0.51 | | | R² = 0.57 | | | R² = 0.74 | | |
|  |  |  |  |  |  |  |  |  |  |  |
| **Specific** | PC1 | 6.63 | 713.86 | *** | 30.22 | 3,058.1 | *** | 139.69 | 4,512.18 | *** |
| **Root length** | PC2 | 6.91 | 3,628.22 | *** | 34.66 | 11,870.42 | *** | 166.82 | 7,385.01 | *** |
|  | PC3 | 6.51 | 181.42 | *** | 28.09 | 1,272.69 | *** | 156.59 | 2,262.44 | *** |
|  | PC4 | 6.71 | 488.37 | *** | 34.81 | 2,263.56 | *** | 163.57 | 1,438.43 | *** |
|  |  | R² = 0.38 | | | R² = 0.41 | | | R² = 0.54 | | |
|  |  |  | | |  | | |  | | |

**Supplementary Table S2 | Original publications for the trait data used.** Listed are the publications that were used to assemble the gap-filled TRY plant trait database^30^ for plant height, specific leaf area, seed mass and specific root length.

| Adler, P. B., Milchunas, D. G., Lauenroth, W. K., Sala, O. E. & Burke, I. C. Functional traits of graminoids in semi-arid steppes: a test of grazing histories. *J. Appl. Ecol*. **41**, 653-663 (2004). |
| --- |
| Adler P. B., Salguero-Gómez R., Compagnoni, A., Hsu, J. S., Ray-Mukherjee, J., Mbeau-Ache, C. & Franco M. Functional traits explain variation in plant life history strategies. *Proc. Natl. Acad. Sci. U.S.A.* **111,** 740-745 (2014). |
| Atkin, O. K. et al. Global variability in leaf respiration in relation to climate, plant functional types and leaf traits. *New Phytol.* **206**, 614-636 (2015). |
| Auger, S. & Shipley, B. Interspecific and intraspecific trait variation along short environmental gradients in an old-growth temperate forest. *J. Veg. Sci.* **24**, 419-428 (2012). |
| Baastrup-Spohr, L., Sand-Jensen, K., Nicolajsen, S. V. & Bruun, H. H. From soaking wet to bone dry: predicting plant community composition along a steep hydrological gradient. *J. Veg. Sci.* **26,** 619-630 (2015). |
| Bahar, N. H. A. et al. Leaf-level photosynthetic capacity in lowland Amazonian and high-elevation Andean tropical moist forests of Peru. *New Phytol.* 214 (3), 1002–1018 (2017). |
| Baraloto, C. et al. Decoupled leaf and stem economics in rainforest trees. *Ecol. Lett.* **13**, 1338-1347 (2010). |
| Baruch, Z. & Goldstein, G. Leaf construction cost, nutrient concentration, and net CO_2_ assimilation of native and invasive species in Hawaii. *Oecologia* **121**, 183-192 (1999). |
| Berner, L. T. et al. Biomass allometry for alder, dwarf birch, and willow in boreal forest and tundra ecosystems of far northeastern Siberia and north-central Alaska. *For. Ecol. Manag.* **337**, 110-118 (2015). |
| Blonder, B., Baldwin, B., Enquist, B.J. & Robichaux, R.H. Variation and macroevolution in leaf functional traits in the Hawaiian silversword alliance (*Asteraceae*). *J. Ecol.* **104**, 219-228 (2016). |
| Blonder, B. et al. The leaf-are shrinkage effect can bias paleoclimate and ecology research. *Am. J. Bot*. **99,** 1756-1763 (2012). |
| Blonder, B., Vasseur, F., Violle, C., Shipley, B., Enquist, B. J. & Vile, D. Testing models for the leaf economics spectrum with leaf and whole-plant traits in Arabidopsis thaliana. *AoB PLANTS* **7**, plv049 (2015). |
| Blonder, B., Violle, C. & Enquist, B. J. Assessing the causes and scales of the leaf economics spectrum using venation networks in *Populus tremuloides*. *J. Ecol* **101**, 981-989 (2013). |
| Blonder, B., Violle, C., Patrick, L. & Enquist, B. Leaf venation networks and the origin of the leaf economics spectrum. *Ecol. Lett.* **14**, 91-100 (2011). |
| Bocanegra-Gonzalez K. T., Fermandez-Mendez F. & Galvis-Jimenez, J. F. (in press) Determinación de la resiliencia en bosques secundarios húmedos tropicales a través de la diversidad funcional de árboles en la región del Bajo Calima, Buenaventura, Colombia. |
| Bond-Lamberty, B., Wang, C. & Gower, S. T. Above- and belowground biomass and sapwood area allometric equations for six boreal tree species of northern Manitoba, *Can. J. For. Res.* **32**, 1441-1450 (2002) |
| Bond-Lamberty, B., Wang, C. & Gower S. T. Leaf area dynamics of a boreal black spruce fire chronosequence. *Tree Physiol.*, **22**, 993-1001 (2002). |
| Boucher, F.C., Thuiller, W., Arnoldi, C., Albert, C.H. & Lavergne, S. Unravelling the architecture of functional variability in wild populations of *Polygonum viviparum* L. *Funct. Ecol.* **27**, 382-391 (2013). |
| Bragazza, L. Conservation priority of Italian alpine habitats: a floristic approach based on potential distribution of vascular plant species. *Biodivers. Conserv.* **18**, 2823-2835 (2009). |
| Choat, B. et al. Global convergence in the vulnerability of forests to drought. *Nature*, **491**, 752-755 (2012). |
| Brown, K. A. et al. Use of provisioning ecosystem services drives loss of functional traits across land use intensification gradients in tropical forests in Madagascar. *Biol. Conserv.* **161**, 118-127, (2013). |
| Buchanan, S., Isaac, M. E., van den Meersche, K. & Martin, A. R. Functional traits of coffee along a shade and fertility gradient in coffee agroforestry systems. *Agrofor. Syst* **93**,1261-1273 (2019). |
| Bucher, S. F. et al. Inter- and intraspecific variation in stomatal pore area index along elevational gradients and its relation to leaf functional traits. *Plant Ecol.* **217**, 229-240 (2016). |
| Burrascano, S. et al. Wild boar rooting intensity determines shifts in understorey composition and functional traits. *Community Ecol.* **16**, 244-253 (2015). |
| Butterfield, B. J. & Briggs, J. M. Regeneration niche differentiates functional strategies of desert woody plant species. *Oecologia* **165**, 477-487 (2011). |
| Byun, C., de Blois, S. & Brisson, J. Plant functional group identity and diversity determine biotic resistance to invasion by an exotic grass. *J. Ecol.* **101**, 128-139 (2013). |
| Cadotte, M. W. Functional traits explain ecosystem function through opposing mechanisms. *Ecol. Lett.* **20**, 989-996 (2017). |
| Campetella, G. et al. Patterns of plant trait-environment relationships along a forest succession chronosequence. *Agric. Ecosyst. Environ.* **145**, 38-48 (2011). |
| Carswell, F. E. et al. Photosynthetic capacity in a central Amazonian rain forest. *Tree physiol.* **20**, 179-186 (2000). |
| Catford, J. A., Morris, W. K., Vesk, P. A., Gippel, C. J. & Downes, B. J. Species and environmental characteristics point to flow regulation and drought as drivers of riparian plant invasion. *Divers. Distrib.* **20**, 1084-1096 (2014). |
| Cavender-Bares, J., Keen, A. & Miles, B. Phylogenetic structure of floridian plant communities depends on taxonomic and spatial scale. *Ecology* **87**, 109-S122 (2006). |
| Cerabolini B., Pierce S., Luzzaro A. & Ossola A. Species evenness affects ecosystem processes in situ via diversity in the adaptive strategies of dominant species. *Plant Ecol.* **207**, 333-345 (2010). |
| Cerabolini B. E. L., Brusa G., Ceriani R. M., De Andreis R., Luzzaro A. & Pierce S. Can CSR classification be generally applied outside Britain? Plant Ecol. **210**, 253-261 (2010). |
| Chacón-Madrigal, E., Wanek, W., Hietz, P. & Dullinger, S. Traits indicating a conservative resource strategy are weakly related to narrow range size in a group of neotropical trees. *Perspect. Plant Ecol.* **32**, 30-37 (2018). |
| Ciccarelli D. Mediterranean coastal dune vegetation: Are disturbance and stress the key selective forces that drive the psammophilous succession? *Estuar. Coast. Shelf Sci.* **165**, 247-253 (2015). |
| Ciocarlan V. The illustrated Flora of Romania. Pteridophyta et Spermatopyta. Edited by Ceres, 1141 (2009). |
| Coomes, D. A., Heathcote, S., Godfrey, E. R., Shepherd, J. J. & Sack L. Scaling of xylem vessels and veins within the leaves of oak species. *Biol. Lett.* **4**, 302-306 (2008). |
| Cornelissen J. H. C. et al. Leaf digestibility and litter decomposability are related in a wide range of subarctic plant species and types. In *Funct. Ecol.* **18**, 779-786 (2004). |
| Cornelissen, J. H. C. An experimental comparison of leaf decomposition rates in a wide range of temperate plant species and types. *J. Ecol.* **84**, 573-582 (1996). |
| Cornelissen, J. H. C. et al. Functional traits of woody plants: correspondence of species rankings between field adults and laboratory-grown seedlings? *J. Veg. Sci.* **14**, 311-322 (2003). |
| Cornelissen, J. H. C., Castro, D. P. & Hunt, R. Seedling growth, allocation and leaf attributes in a wide range of woody plant species and types. *J. Ecol.* **84**, 755-765 (1996). |
| Cornwell, W. K., Bhaskar, R., Sack, L., Cordell, S. & Lunch, C. K. Adjustment of structure and function of Hawaiian *Metrosideros polymorpha* at high vs. low precipitation. *Funct. Ecol.* **21**, 1063-1071 (2007). |
| Craine J. M. et al. Functional consequences of climate-change induced plant species loss in a tallgrass prairie. *Oecologia* **165**, 1109-1117 (2011). |
| Dahlin K. M., Asner G. P. & Field, C. B. Environmental and community controls on plant canopy chemistry in a Mediterranean-type ecosystem. *Proc. Natl. Acad. Sci. U.S.A.* **110,** 6895-6900 (2013). |
| Dalke, I. V., Novakovskiy, A. B., Maslova, S. P. & Dubrovskiy, Y. A. Morphological and functional traits of herbaceous plants with different functional types in the European Northeast. *Plant Ecol.* **219**, 1295-1305 (2018). |
| Dang-Le, A. T., Edelin, C. & Le-Cong, K. Ontogenetic variations in leaf morphology of the tropical rain forest species *Dipterocarpus alatus* Roxb. ex G. Don. *Trees* **27**, 773-786 (2013). |
| Dawson, S. K. et al. Plant traits of propagule banks and standing vegetation reveal flooding alleviates impacts of agriculture on wetland restoration. *J. Appl. Ecol.* **54**, 1907-1918 (2017). |
| de Frutos A., Navarro, T., Pueyo, Y., Alados, C. L. Inferring resilience to fragmentation-induced changes in plant communities in a semi-arid Mediterranean ecosystem. *PloS one* **10**, e0118837 (2015). |
| Diaz, S. et al. The plant traits that drive ecosystems: evidence from three continents. *J. Veg. Sci.* **15**, 295-304 (2004). |
| Domingues T. F. et al. Co-limitation of photosynthetic capacity by nitrogen and phosphorus in West Africa woodlands. *Plant, Cell Environ.* **33**, 959-980 (2010). |
| Dunbar-Co, S., Sporck, M. J. & Sack L. Leaf trait diversification and design in seven rare taxa of the Hawaiian *Plantago* radiation. *Int. J. Plant Sci.* **170**, 61-75 (2009). |
| Dwyer, J. M., Hobbs, R. J. & Mayfield, M. M. Specific leaf area responses to environmental gradients through space and time. *Ecology* **95**, 399-410 (2014). |
| Fagúndez, J. & Izco, J. Seed morphology of two distinct species of *Erica* L. (*Ericaceae*). *Acta Bot. Malacit.* **33**, 1-9 (2008). |
| Falster, D. S. et al. BAAD: a Biomass and allometry database for woody plants. *Ecology* **96** , 1445-1445 (2015). |
| Fitter, A. H. & Peat, H. J. The Ecological Flora Database. *J. Ecology*, **82**, 415-425 (1994). |
| Fonseca, C. R., Overton, J. M., Collins, B. & Westoby, M. Shifts in trait-combinations along rainfall and phosphorus gradients. *J. Ecol.* **88**, 964-977 (2000). |
| Forgiarini, C., Souza, A. F., Longhi, S. J., Oliveira, J. M. In the lack of extreme pioneers: trait relationships and ecological strategies of 66 subtropical tree species. *J. Plant Ecol.* **8**, 359-367 (2015). |
| Frenette-Dussault, C., Shipley, B., Léger, J. F., Meziane, D. & Hingrat, Y. Functional structure of an arid steppe plant community reveals similarities with Grime's C-S-R theory. *J. Veg. Sci.* **23**, 208-222 (2012). |
| Freschet, G. T., Cornelissen, J. H. C., van Logtestijn, R. S. P. & Aerts, R. Evidence of the ‘plant economics spectrum’ in a subarctic flora. *J. Ecol.* **98**, 362-373 (2010). |
| Fyllas, N. M. et al. Basin-wide variations in foliar properties of Amazonian forest: phylogeny, soils and climate. *Biogeosciences* **6**, 2677-2708 (2009). |
| Gachet, S., Véla, E., Tatoni, T. BASECO: a floristic and ecological database of Mediterranean French flora. *Biodivers. Conserv.* **14**, 1023-1034 (2005). |
| Garnier, E. et al. Assessing the effects of land-use change on plant traits, communities and ecosystem functioning in grasslands: a standardized methodology and lessons from an application to 11 European sites. *Ann. Bot.* **99**, 967-985 (2007). |
| Giarrizzo, E. et al. Re-visiting historical semi-natural grasslands in the Apennines to assess patterns of changes in species composition and functional traits. *Appl. Veg. Sci.* **20**, 247-258 (2017). |
| Givnish T. J., Montgomery, R. A. & Goldstein, G. Adaptive radiation of photosynthetic physiology in the Hawaiian lobeliads: light regimes, static light responses, and whole-plant compensation points. *Am. J. Bot.* **91**, 228-246 (2004). |
| Gonzalez-Akre, E., McShea, W., Bourg, N. & Anderson-Teixeira, K. Leaf traits data (SLA) for 56 woody species at the Smithsonian Conservation Biology Institute-ForestGEO Forest Dynamic Plot. Front Royal, Virginia. USA. [Data set] (2015). |
| Gos P. et al. Relative contribution of soil, management and traits to co-variations of multiple ecosystem properties in grasslands. *Oecologia* **180**, 1001-1013 (2016). |
| Green, W. USDA PLANTS Compilation. Version 1. National Plant Data Center: Baton Rouge, LA 70874-74490 USA (2009). |
| Gutiérrez A. G. & Huth, A. Successional stages of primary temperate rainforests of Chiloé Island, Chile. *Perspect. Plant Ecol. Evol.* **14**, 243-256 (2012). |
| Guy, A., Mischkolz, Jenalee, M. & Lamb, E. Limited effects of simulated acidic deposition on seedling survivorship and root morphology of endemic plant taxa of the Athabasca sand dunes in well-watered greenhouse trials. *Botany* **91**, 176-181 (2013). |
| Han, W., Fang, J., Guo, D. & Zhang, Y. Leaf nitrogen and phosphorus stoichiometry across 753 terrestrial plant species in China. *New Phytol.* **168**, 377-385 (2005). |
| Hao, G. Y., Sack, L., Wang, A. Y., Cao, K. F. & Goldstein, G. Differentiation of leaf water flux and drought tolerance traits in hemiepiphytic and non-hemiepiphytic *Ficus* tree species. *Funct. Ecol.* **24**, 731-740 (2010). |
| Hattermann, D., Elstner, C., Bernhardt-Römermann, M. & Eckstein, L. Measurements from the project „Relative effects of local and regional factors as drivers for plant community diversity, functional trait diversity and genetic structure of species on Baltic uplift islands” funded by the German Research Foundation - DFG: BE 4143/5-1 and EC 209/12-1 (2014-2018). |
| Herz, K., Dietz, S., Haider, S., Jandt, U., Scheel, D. & Bruelheide, H. Drivers of intraspecific trait variation of grass and forb species in German meadows and pastures. *J. Veg. Sci.* **28**, 705-716 (2017). |
| Hickler, T. Plant functional types and community characteristics along environmental gradients on Öland's Great Alvar (Sweden) Master Thesis, University of Lund, Sweden (1999). |
| Higuchi, P. & Silva, A. C. Araucaria Forest Database (2013). |
| Hill, M.O., Preston, C. D. & Roy, D. B. PLANTATT - attributes of British and Irish Plants: status, size, life history, geography and habitats. Huntingdon: Centre for Ecology and Hydrology (2004). |
| Hoof, J., Sack, L., Webb, D. T. & Nilsen, E. T. Contrasting structure and function of pubescent and glabrous varieties of Hawaiian *Metrosideros polymorpha* (Myrtaceae) at high elevation. *Biotropica* **40**, 113-118 (2008). |
| Iversen, C. M. et al. A global fine-root ecology database to address below-ground challenges in plant ecology. *New Phytol.* **215**, 15-26 (2017). |
| Powers, J. S. & Tiffin, P. Plant functional type classifications in tropical dry forests in Costa Rica: leaf habit versus taxonomic approaches. *Funct. Ecol.* **24**, 927-936 (2010). |
| Joseph, G. S., Seymour, C. L., Cumming, G. S., Cumming, D. H. M., & Mahlangu, Z. Termite mounds increase functional diversity of woody plants in African savannas. *Ecosyst.* **17**, 808-819 (2014). |
| Kattenborn, T., Fassnacht, F. E. & Sebastian, S. Differentiating plant functional types using reflectance: which traits make the difference? *Remote Sens. Ecol. Conserv.* **5**, 5-19 (2019). |
| Kattge, J., Knorr, W., Raddatz, T. & Wirth, C. Quantifying photosynthetic capacity and its relationship to leaf nitrogen content for global-scale terrestrial biosphere models. *Glob. Chang. Biol.* **15**, 976-991 (2009). |
| Kearsley, E. et al. Functional community structure of African monodominant *Gilbertiodendron dewevrei* forest influenced by local environmental filtering. *Ecol. Evol.* **7**, 295-304 (2017). |
| Kichenin, E., Wardle, D. A., Peltzer, D. A., Morse, C. W. & Freschet, G. T. Contrasting effects of plant inter- and intraspecific variation on community-level trait measures along an environmental gradient. *Funct. Ecol.* **27**, 1254-1261 (2013) |
| Kirkup, D., Malcom, P., Christian, G. & Paton, A. Towards a digital African flora. *Taxon* **54**, 457-466 (2005). |
| Kleyer, M. et al. The LEDA Traitbase: a database of life-history traits of the Northwest European flora. *J. Ecol.* **96**, 1266-1274 (2008). |
| Knauer, J. et al. Towards physiologically meaningful water-use efficiency estimates from eddy covariance data. *Glob. Chang. Biol.* **24**, 694-710 (2018). |
| Koike, F. Plant traits as predictors of woody species dominance in climax forest communities. *J. Veg. Sci.* **12**, 327-336 (2001). |
| Komac, B., Pladevall, C., Domenech, M. & Fanlo, R. Functional diversity and grazing intensity in sub-alpine and alpine grasslands in Andorra. *Appl. Veg. Sci.* **18**, 75-85 (2014). |
| Kraft, N. J. B., Valencia, R. & Ackerly, D. Functional traits and niche-based tree community assembly in an Amazonian forest. *Science* **322**, 580-582 (2008). |
| Kühn, I., Durka, W. & Klotz, S. BiolFlor - a new plant-trait database as a tool for plant invasion ecology. *Divers. Distrib.* **10**, 363-365 (2004). |
| Kurokawa, H. & Nakashizuka, T. Leaf herbivory and decomposability in a Malaysian tropical rain forest. *Ecology* **89**, 2645-2656 (2008). |
| La Pierre, K. J. & Smith, M. D. Functional trait expression of grassland species shift with short- and long-term nutrient additions. *Plant Ecol.* **216**, 307-318 (2015). |
| Laughlin, D. C., Leppert, J. J., Moore, M. M. & Sieg, C. H. A multi-trait test of the leaf-height-seed plant strategy scheme with 133 species from a pine forest flora. *Funct. Ecol.* **24**, 493-501 (2010). |
| Laughlin, D. C., Fulé, P. Z., Huffman, D. W., Crouse, J. & Laliberte, E. Climatic constraints on trait-based forest assembly. *J. Ecol.* **99**, 1489-1499 (2011). |
| Lhotsky, B., Csecserits, A., Kovács, B., Botta-Dukát, Z. New plant trait records of the Hungarian flora. *Acta Bot. Hung.* **58**, 397-400 (2016). |
| Li, R. et al. Are functional traits a good predictor of global change impacts on tree species abundance dynamics in a subtropical forest? *Ecol. Lett.* **18**, 1181-1189 (2015). |
| Li, Y. & Shipley, B. Community divergence and convergence along experimental gradients of stress and disturbance. *Ecology* **99**, 775-781 (2018). |
| Liebergesell, M. et al. Functional resilience against climate-driven extinctions - comparing the functional diversity of European and North American tree floras. *PloS one* **11**, e0148607 (2016). |
| Lin, Y.-S. et al. Optimal stomatal behaviour around the world. *Nat. Clim. Change* **5**, 459-464 (2015). |
| Louault, F., Pillar, V. D., Aufrere, J., Garnier, E. & Soussana, J. F. Plant traits and functional types in response to reduced disturbance in a semi-natural grassland. *J. Veg. Sci.* **16**, 151-160 (2005). |
| Lukeš, P., Stenberg, P., Rautiainen, M., Mõttus, M. & Vanhatalo, K.M. Optical properties of leaves and needles for boreal tree species in Europe. *Remote Sens. Lett.*, **4**, 667-676 (2013). |
| Maire, V. et al. Global effects of soil and climate on leaf photosynthetic traits and rates. *Glob. Ecol. Biogeo.* **24**, 706-717 (2015). |
| Moretti, M. & Legg, C. Combining plant and animal traits to assess community functional responses to disturbance. *Ecography* **32**, 299-309 (2009). |
| Martin A. R. et al. Inter- and intraspecific variation in leaf economics traits in wheat and maize, *AoB Plants*, ply006 (2018). |
| Martin, R. E., Asner, G. P. & Sack, L. Genetic variation in leaf pigment, optical and photosynthetic function among diverse phenotypes of *Metrosideros polymorpha* grown in a common garden. *Oecologia* **151**, 387-400 (2007). |
| Martinez-Garza, C., Bongers, F. & Poorter, L. Are functional traits good predictors of species performance in restoration plantings in tropical abandoned pastures? *For. Ecol. Manag.* **303**, 35-45 (2013). |
| McPartland M. Alaska Peatland Experiment (APEX) 2016 PFT values |
| Medeiros, J. S., Burns, J. H., Nicholson, J., Rogers, L., & Valverde-Barrantes, O. Decoupled leaf and root carbon economics is a key component in the ecological diversity and evolutionary divergence of deciduous and evergreen lineages of genus *Rhododendron*. *Am. J. Bot.* **104**, 803-816 (2017). |
| Medlyn, B. E. et al. Effects of elevated CO_2_ on photosynthesis in European forest species: a meta-analysis of model parameters. *Plant Cell Environ.* **22**, 1475-1495 (1999). |
| Meir, P. & Levy, P. E. Photosynthetic parameters from two contrasting woody vegetation types in West Africa. Plant Ecology. **192**, 277-287 (2007). |
| Meir, P., Kruijt, B., Broadmeadow, M., Kull, O., Carswell, F., Nobre, A. & Jarvis, P. G. Acclimation of photosynthetic capacity to irradiance in tree canopies in relation to leaf nitrogen concentration and leaf mass per unit area. *Plant Cell Environ.* **25**, 343-357 (2002). |
| Mencuccini, M. The ecological significance of long distance water transport: short-term regulation and long-term acclimation across plant growth forms. *Plant Cell Environ.* **26**, 163-182 (2003). |
| Messier, J., McGill, B. J. & Lechowicz, M. J. How do traits vary across ecological scales? A case for trait-based ecology. *Ecol. Lett.* **13**, 838-848 (2010). |
| Michaletz, S.T. & Johnson, E.A. A heat transfer model of crown scorch in forest fires. *Can. J. For. Res.* **36**, 2839-2851 (2006) |
| Milla, R. & Reich, P. B. Multi-trait interactions, not phylogeny, fine-tune leaf size reduction with increasing altitude. *Ann. Bot.* **107**, 455-465 (2011). |
| Miller, J. E. D., Ives, A. R., Harrison, S. P. & Damschen E. I. Early and late flowering guilds respond differently to landscape spatial structure. *J. Ecol.* **106**, 1033-1045 (2018). |
| Minden V. & Kleyer, M. Ecosystem multifunctionality of coastal marshes is determined by key plant traits. *J. Veg. Sci.* **26**, 651-662 (2015). |
| Minden, V. & Kleyer, M. Testing the effect–response framework: key response and effect traits determining above-ground biomass of salt marshes. *J. Veg. Sci.* **22**, 387-401 (2011). |
| Minden, V., Andratschke, S., Spalke, J., Timmermann, H. & Kleyer, M. Plant trait–environment relationships in salt marshes: deviations from predictions by ecological concepts. *Perspect. Plant Ecol.* **14**, 183-192 (2012). |
| Moles, A. T., Falster, D. S., Leishman, M. R. & Westoby, M. Small-seeded species produce more seeds per square metre of canopy per year, but not per individual per lifetime. *J. Ecol.* **92**, 384-396 (2004). |
| Mori, A. S. et al. Functional redundancy of multiple forest taxa along an elevational gradient: Predicting the consequences of non-random species loss. *J. Biogeo.* **42**, 1383-1396 (2015). |
| Muller, S. C., Overbeck, G. E., Pfadenhauer, J. & Pillar, V. D. Plant functional types of woody species related to fire disturbance in forest-grassland ecotones. *Plant Ecol.* **189**, 1-14 (2007). |
| Neuschulz, E. L., Mueller, T., Schleuning, M. & Böhning-Gaese, K. Pollination and seed dispersal are the most threatened processes of plant regeneration. *Sci. Rep.* **6**, 29839 (2016). |
| Niinemets, U. Global-scale climatic controls of leaf dry mass per area, density, and thickness in trees and shrubs. *Ecology* **82**, 453-469 (2001). |
| Nolan, R.H., Fairweather, K.A., Tarin, T., Santini, N.S., Cleverly, J., Faux, R. & Eamus, D. Divergence in plant water-use strategies in semiarid woody species. *Funct. Plant Biol.* **44**, 1134-1146 (2017). |
| Ogaya, R. & Penuelas, J. Comparative field study of *Quercus ilex* and *Phillyrea latifolia*: photosynthetic response to experimental drought conditions. *Environ. Exp. Bot.* **50**, 137-148 (2003). |
| Onoda, Y. et al. Global patterns of leaf mechanical properties. *Ecol. Lett.* **14**, 301-312 (2011). |
| Onoda, Y. et al. Physiological and structural tradeoffs underlying the leaf economics spectrum. *New Phytol.* **214**, 1447-1463 (2017). |
| Onstein, R. E., Carter, R. J., Xing, Y. & Linder, H. P. Diversification rate shifts in the Cape Floristic Region: The right traits in the right place at the right time. *Perspect. Plant Ecol.* **16**, 331-340 (2014). |
| Ordonez, J. C., van Bodegom, P. M., Witte, J. P. M., Bartholomeus, R. P., van Hal, J. R. & Aerts, R. Plant strategies in relation to resource supply in mesic to wet environments: Does theory mirror nature? *Am. Nat.* **175**, 225-239 (2010). |
| Pahl, A.T., Kollmann, J., Mayer, A. & Haider, S. No evidence for local adaptation in an invasive alien plant: field and greenhouse experiments tracing a colonization sequence. *Ann. Bot.* **112**, 1921-1930 (2013). |
| Paine, C. E. T. et al. Globally, functional traits are weak predictors of juvenile tree growth, and we do not know why. *J. Ecol.* **103**, 978-989 (2015). |
| Paula, S. et al. Fire-related traits for plant species of the Mediterranean Basin. *Ecology* **90**, 1420 (2009). |
| Peco B., de Pablos, I., Traba, J. & Levassor C. The effect of grazing abandonment on species composition and functional traits: the case of dehesa grasslands. *Basic Appl. Ecol.* **6**, 175-183 (2005). |
| Petter, G. et al. Functional leaf traits of vascular epiphytes: vertical trends within the forest, intra‐ and interspecific trait variability, and taxonomic signals. *Funct. Ecol.* **30**, 188-198 (2016). |
| Pierce S., Brusa G., Sartori M. & Cerabolini B.E.L. Combined use of leaf size and economics traits allows direct comparison of hydrophyte and terrestrial herbaceous adaptive strategies. *Ann. Bot.* **109**, 1047-1053 (2012). |
| Pierce S., Brusa G., Vagge I. & Cerabolini B.E.L. Allocating CSR plant functional types: the use of leaf economics and size traits to classify woody and herbaceous vascular plants. *Funct. Ecol.* **27**, 1002-1010 (2013). |
| Pierce S., Ceriani R.M., De Andreis R., Luzzaro A. & Cerabolini B. The leaf economics spectrum of *Poaceae* reflects variation in survival strategies. *Plant Biosyst.* **141**, 337-343 (2007). |
| Pierce S., Luzzaro A., Caccianiga M., Ceriani R. M. & Cerabolini B. Disturbance is the principal α-scale filter determining niche differentiation, coexistence and biodiversity in an alpine community. *J. Ecol.* **95**: 698-706 (2007). |
| Pierce S., Vagge I., Brusa G. & Cerabolini B.E.L. The intimacy between sexual traits and Grime’s CSR strategies for orchids coexisting in semi-natural calcareous grassland at the Olive Lawn. *Plant Ecol.* **215**, 495-505 (2014). |
| Pillar, V. D. & Sosinski, E. E. An improved method for searching plant functional types by numerical analysis. *J. Veg. Sci.* **14**, 323-332 (2003). |
| Poorter, H., Niinemets, U., Poorter, L., Wright, I. J. & Villar, R. Causes and consequences of variation in leaf mass per area (LMA): a meta-analysis. *New Phytol.* **182**, 565-588 (2009). |
| Prentice, I.C., Meng, T., Wang, H., Harrison, S.P., Ni, J. & Wang, G. Evidence for a universal scaling relationship of leaf CO_2_ drawdown along a moisture gradient. *New Phytol.* **190**, 169-180 (2011). |
| Preston, K. A., Cornwell, W. K. & Denoyer, J. L. Wood density and vessel traits as distinct correlates of ecological strategy in 51 California coast range angiosperms. *New Phytol.* **170**, 807-818 (2006). |
| Price, C. A. & Enquist, B. J. Scaling of mass and morphology in Dicotyledonous leaves: an extension of the WBE model. *Ecology* **88**, 1132-1141 (2007). |
| Pyankov, V. I., Kondratchuk, A. V. & Shipley, B. Leaf structure and specific leaf mass: the alpine desert plants of the Eastern Pamirs, Tadjikistan. *New Phytol.* **143**, 131-142 (1999). |
| Quested, H. M. et al. Decomposition of sub-arctic plants with differing nitrogen economies: a functional role for hemiparasites. *Ecology* **84**, 3209-3221 (2003). |
| Reich, P. B., Oleksyn, J. & Wright, I. J. Leaf phosphorus influences the photosynthesis-nitrogen relation: a cross-biome analysis of 314 species. *Oecologia* **160**, 207-212 (2009). |
| Reich, P. B., Tjoelker, M. G., Pregitzer, K. S., Wright, I. J., Oleksyn, J. & Machado, J. L. Scaling of respiration to nitrogen in leaves, stems and roots of higher land plants. *Ecol. Lett.* **11**, 793-801 (2008). |
| Rodrigues, A.V., Bones, F.L.V., Schneiders, A., Oliveira, L.Z., Vibrans, A.C., Gasper, A.L. Plant trait dataset for tree-like growth forms species of the subtropical Atlantic rain forest in Brazil. *Data* **3**, 16 (2018). |
| Rogers, A., Serbin, S. P., Ely, K. S., Sloan, V., L. & Wullschleger, S. D. Terrestrial biosphere models underestimate photosynthetic capacity and CO_2_ assimilation in the Arctic. *New Phytol.* **216**, 1090-1103 (2017). |
| Rolo, V., Olivier, P. & van Aarde, R. Seeded pioneer die-offs reduce the functional trait space of new-growth coastal dune forests. For. Ecol. Manag. **377**, 26-35 (2016). |
| Royal Botanical Gardens KEW. Seed Information Database (SID). Version 7.1. http://data.kew.org/sid. Accessed in May 2008. |
| Royal Botanical Gardens KEW. Seed Information Database (SID) Version 7.1. http://data.kew.org/sid. Accessed in May 2014. |
| Sack, L. Responses of temperate woody seedlings to shade and drought: do trade-offs limit potential niche differentiation? *Oikos* **107**, 110-127 (2004). |
| Sack, L., Tyree, M. T. & Holbrook, N. M. Leaf hydraulic architecture correlates with regeneration irradiance in tropical rainforest trees. *New Phytol.* **167**, 403-413 (2005). |
| Sack, L., Cowan, P. D., Jaikumar, N. & Holbrook, N. M. The 'hydrology' of leaves: co-ordination of structure and function in temperate woody species. *Plant Cell Environ.* **26**, 1343-1356 (2003). |
| Sack, L., Melcher, P. J., Liu, W. H., Middleton, E. & Pardee, T. How strong is intracanopy leaf plasticity in temperate deciduous trees? *Am. J. Bot.* **93**, 829-839 (2006). |
| Sandel, B., Corbin, J. D. & Krupa, M. Using plant functional traits to guide restoration: a case study in California coastal grassland. *Ecosphere* **2**, 1-16 (2011). |
| Scalon, M. C., Haridasan, M., Franco, A. C. Influence of long-term nutrient manipulation on specific leaf area and leaf nutrient concentrations in savanna woody species of contrasting leaf phenologies. *Plant Soil* **421**, 233-244 (2017). |
| Scherer-Lorenzen, M., Schulze, E., Don, A., Schumacher, J., Weller, E. Exploring the functional significance of forest diversity: A new long-term experiment with temperate tree species (BIOTREE). *Percept. Plant Ecol.* **9**, 53-70 (2007). |
| Schurr, F. M., Midgley, G.F., Rebelo, A.G., Reeves, G., Poschlod, P. & Higgins, S. I. *Glob. Ecol. Biogeogr.* **16**, 449-459 (2007). |
| Schweingruber, F. H., Landolt, W. The Xylem Database. Swiss Federal Research Institute WSL (Updated 2005). |
| Scoffoni, C., Pou, A., Aasamaa, K. & Sack, L. The rapid light response of leaf hydraulic conductance: new evidence from two experimental methods. *Plant Cell Environ.* **31**, 1803-1812 (2008). |
| Sharpe, J. M. & Solano, N. Traits of fertile (spore-bearing) leaves of understory rainforest ferns from the El Verde Field Station in the El Yunque National Forest, Puerto Rico, USA. Unpublished data (2016). |
| Sharpe, J. M., Solano, N. Traits of sterile (non-spore bearing) leaves of understory rainforest ferns from the El Verde Field Station in the El Yunque National Forest, Puerto Rico, USA and from the Monteverde cloud forest in Monteverde, Costa Rica. Unpublished data (2016). |
| Shiodera, S., Rahajoe, J. S. & Kohyama, T. Variation in longevity and traits of leaves among co-occurring understorey plants in a tropical montane forest. *J. Trop. Ecol.* **24**, 121-133 (2008). |
| Shipley, B. Structured interspecific determinants of specific leaf-area in 34 species of herbaceous Angiosperms. *Funct. Ecol.* **9**, 312-319 (1995). |
| Shipley, B. & Vu, T. T. Dry matter content as a measure of dry matter concentration in plants and their parts. *New Phytol.* **153**, 359-364 (2002). |
| Siefert, A. Spatial patterns of functional divergence in old-field plant communities. *Oikos* **121**, 907-914 (2012). |
| Siefert, A., Fridley, J. D. & Ritchie, M. E. Community functional responses to soil and climate at multiple spatial scales: when does intraspecific variation matter? *PloS one* **9**, e111189 (2014). |
| Slot, M., Rey-Sanchez, C., Winter, K. & Kitajima, K. Trait-based scaling of temperature-dependent foliar respiration in a species-rich tropical forest canopy. *Funct. Ecol.* **28**, 1074-1086 (2014). |
| Smith, N. G., Dukes, J. S. LCE: leaf carbon exchange data set for tropical, temperate, and boreal species of North and Central America. *Ecology* **98**, 2978-2978 (2017). |
| Smith, S. W., Woodin, S. J., Pakeman, R. J., Johnson, D. & van der Wal, R. Root traits predict decomposition across a landscape-scale grazing experiment. *New Phytol* **203**, 851-862 (2014). |
| Souza, K. et al. Partição de nicho por grupos funcionais de espécies arbóreas em uma floresta subtropical. *Rodriguésia* **68**, 1165-1175 (2017). |
| Spasojevic, M. J. & Suding, K. N. Inferring community assembly mechanisms from functional diversity patterns: the importance of multiple assembly processes. *J. Ecol.* **100**, 652-661 (2012). |
| Spasojevic, M. J., Turner, B. L., Myers, J. A. When does intraspecific trait variation contribute to functional beta‐diversity? *J. Ecol.* **104**, 487-496 (2016). |
| Swaine, E. K. Ecological and evolutionary drivers of plant community assembly in a Bornean rain forest. PhD Thesis, University of Aberdeen, Aberdeen (2007). |
| Swenson, N.G., Anglada-Cordero, P. & Barone, J. A. Deterministic tropical tree community turnover: evidence from patterns of functional beta diversity along an elevational gradient. *Proc. R. Soc. B: Biol. Sci.* **278**, 877-884 (2011). |
| Takkis, K. Changes in plant species richness and population performance in response to habitat loss and fragmentation. PhD Thesis, Universitatis Tartuensis, (2014). |
| Takkis, K., Saar, L., Pärtel, M., Helm, A. Effect of environment and landscape on the traits of six plant species in fragmented grasslands (in preparation). |
| Thuiller W. Traits of European Alpine Flora - OriginAlps Project - Centre National de la Recherche Scientifique. |
| Domingues, T. F., Martinelli, L. A. & Ehleringer, J. R. Ecophysiological traits of plant functional groups in forest and pasture ecosystems from eastern Amazonia, Brazil. *Plant Ecol.* **193**, 101-112 (2007). |
| Tribouillois, H. et al. A Functional characterisation of a wide range of cover crop species: growth and nitrogen acquisition rates, leaf traits and ecological strategies. *PloS one* **10**, e0122156 (2015). |
| van de Weg, M. J., Meir, P., Grace, J. & Atkin, O. Altitudinal variation in leaf mass per unit area, leaf tissue density and foliar nitrogen and phosphorus content along the Amazon-Andes gradient in Peru. *Plant Ecol. Divers.* **2**, 243-254 (2009). |
| van de Weg, M. J., Meir, P., Grace, J., Ramos, G. D. Photosynthetic parameters, dark respiration and leaf traits in the canopy of a Peruvian tropical montane cloud forest. *Oecologia* **168**, 23-34 (2011) |
| van der Plas, F. & Olff, H. Mesoherbivores affect grasshopper communities in a megaherbivore-dominated South African savannah. *Oecologia* **175**, 639-649 (2014). |
| Vergutz, L., Manzoni, S., Porporato, A., Novais, R. F. & Jackson, R.B. A global database of carbon and nutrient concentrations of green and senesced leaves. Data set from Oak Ridge National Laboratory. http://daac.ornl.gov (2012). |
| Vile, D. Significations fonctionnelle et ecologique des traits des especes vegetales: exemple dans une succession post-cultural mediterraneenne et generalisations. PHD Thesis, Université de Sherbrooke (Québec, Canada) (2005). |
| Von Holle, B. & Simberloff, D. Testing Fox's assembly rule: Does plant invasion depend on recipient community structure? *Oikos* **105**, 551-563 (2004). |
| Waite, M. & Sack, L. How does moss photosynthesis relate to leaf and canopy structure? Trait relationships for 10 Hawaiian species of contrasting light habitats. *New Phytol.* **185**, 156-172 (2010). |
| Walker, A.P. A global data set of leaf photosynthetic rates, leaf N and P, and specific leaf area. Data set. Available from Oak Ridge National Laboratory (http://daac.ornl.gov), Oak Ridge, Tennessee, USA (2014). |
| Wang, H. et al. The China Plant Trait Database. Edited by Pangea. https://doi.pangaea.de/10.1594/PANGAEA.871819 (2017). |
| Williams, M., Shimabokuro, Y.E. & Rastetter, E.B. LBA-ECO CD-09 soil and vegetation characteristics, Tapajos National Forest, Brazil. Data set. Available from Oak Ridge National Laboratory (http://daac.ornl.gov), Oak Ridge, Tennessee, USA (2012). |
| Willis, C. G. et al. Phylogenetic community structure in Minnesota oak savanna is influenced by spatial extent and environmental variation. *Ecography* **33**, 565-577 (2010). |
| Wilson K., Baldocchi, D., Hanson, P. Spatial and seasonal variability of photosynthetic parameters and their relationship to leaf nitrogen in a deciduous forest. *Tree Physiol.* **20**, 565-578 (2000). |
| Wirth, C. & Lichstein, J. W. The imprint of species turnover on old-growth forest carbon balances - Insights from a trait-based model of forest dynamics. in Old-Growth Forests 81-113, eds. Wirth, C., Gleixner, G. & Heimann, M. (2009). |
| Wright, I. J. et al. Relationships among ecologically important dimensions of plant trait variation in seven Neotropical forests. *Ann. Bot.* **99**, 1003-1015 (2007). |
| Wright, I. J. et al. The worldwide leaf economics spectrum. *Nature* **428**, 821-827 (2004). |
| Wright, S. J. et al. Functional traits and the growth–mortality trade-off in tropical trees. *Ecology* **91**, 3664-3674 (2010). |
| Zheng, W. Silva Sinica: Volume 1-4. China Forestry Publishing House, Beijing (1983). |


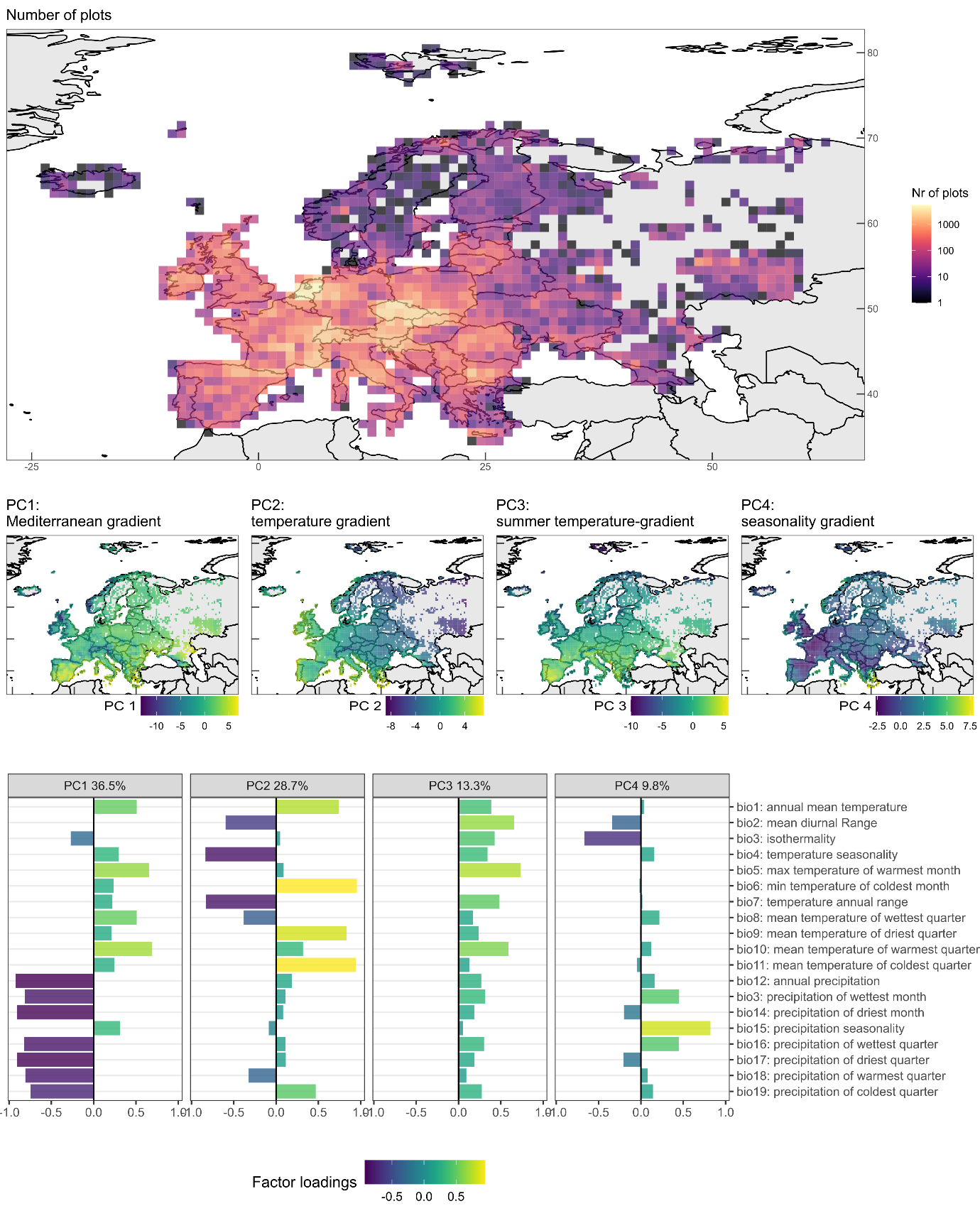


**Supplementary Fig. S1 | Distribution and principal component scores of the 300,021 plots.** Top panel: Number of plots on a log10 scale, aggregated to 1 x 1 degrees of longitude and latitude. Middle panel: Plot-level values of the four principal components (PCs) from the 19 bioclimatic variables, averaged at a resolution of 0.5 x 0.5 degrees of longitude and latitude. Bottom panel: Factor loadings from the principal component analysis of the 19 bioclimatic variables along the four principal components. Percentages boxes represent the captured proportion of variation in the bioclimatic variables.


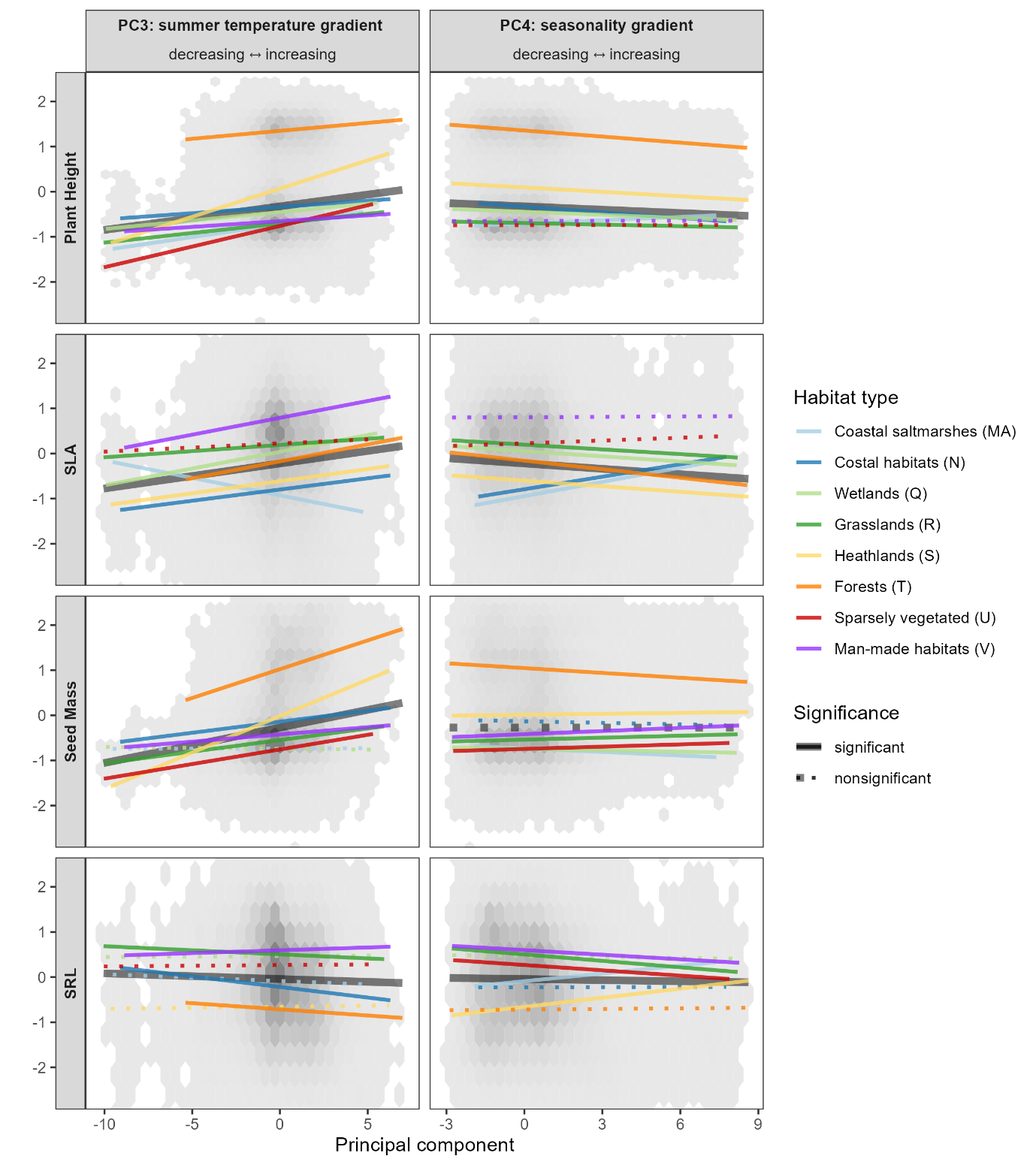


**Supplementary Fig. S2 | Effects of climate on four plant traits in broad habitats.** The graphs show the community-weighted means of four plant functional traits as linear functions of the **third** and **fourth** principal components (PCs) of the 19 CHELSA bioclimatic variables, obtained with mixed-effects additive models. Slopes show the relationships across all (black) and within the most broadly defined habitats of the EUNIS classification (colours). Solid lines indicate significant relationships at p < 0.05 (based on separate two-sided t-tests). Grey hexagons show the distribution of plot-level observations. SLA: specific leaf area, SRL: specific root length.


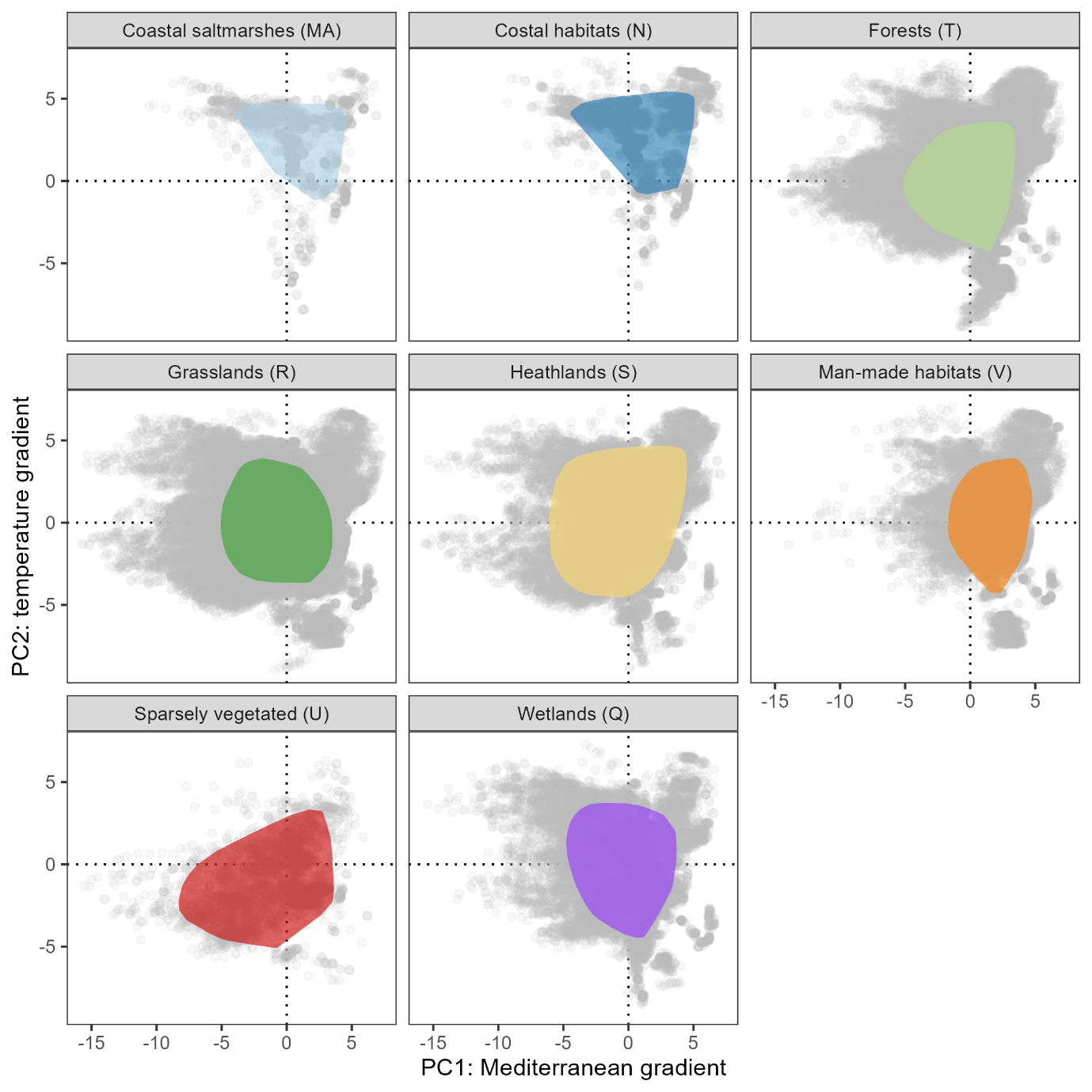


**Supplementary Fig. S3 | Distribution of the eight broad EUNIS level 1 habitats along the first and second principal components (PCs) of the 19 CHELSA bioclimatic variables**. The grey points show the position of the individual plots. The coloured polygons show the position of the minimum convex hulls that cover 80% of the plots. The colours refer to the different EUNIS habitats.


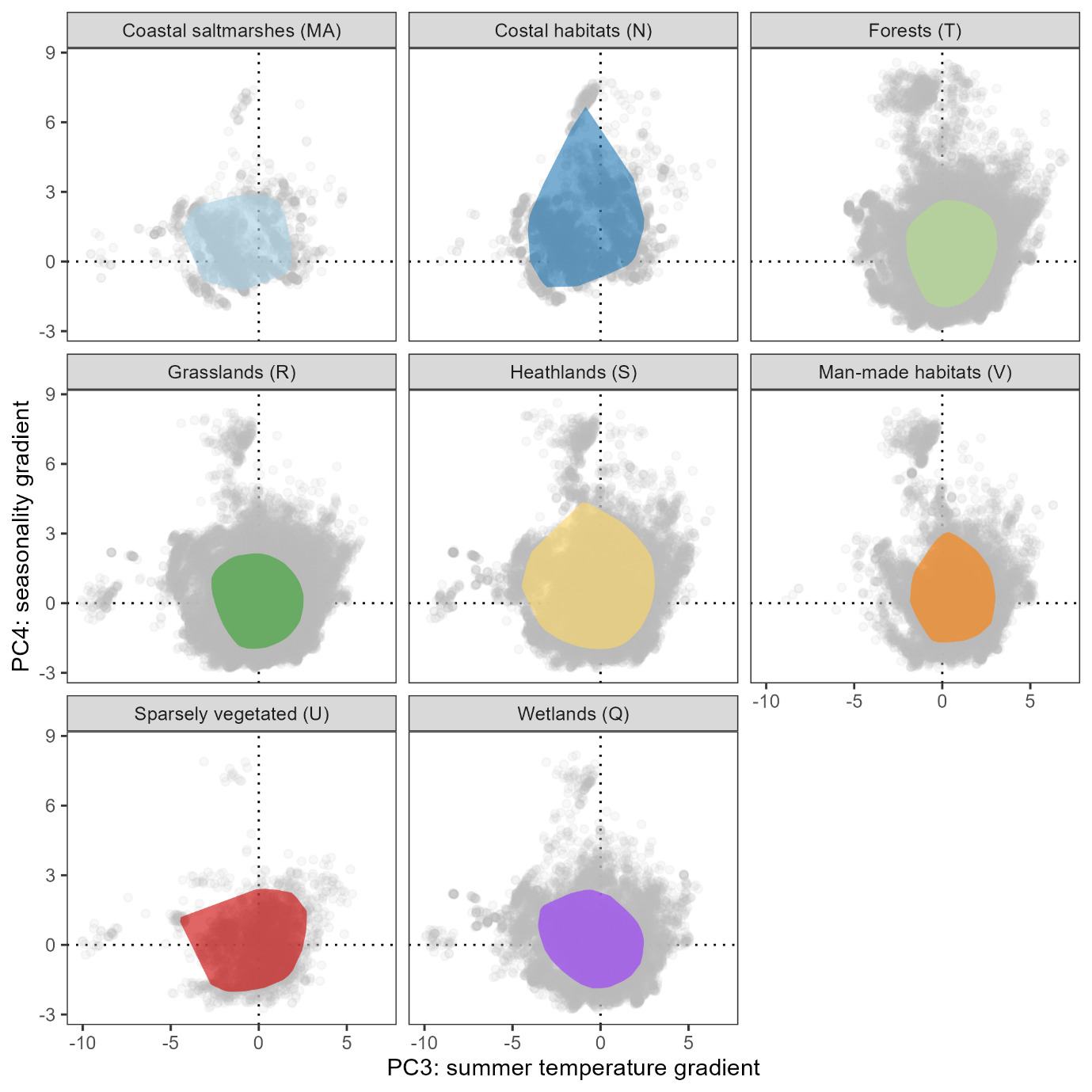


**Supplementary Fig. S4 | Distribution of the eight broad EUNIS level 1 habitats along the third and fourth principal components (PCs) of the 19 CHELSA bioclimatic variables**. The grey points show the position of the individual plots. The coloured polygons show the position of the minimum convex hulls that cover 80% of the plots. The colours refer to the different EUNIS habitats.


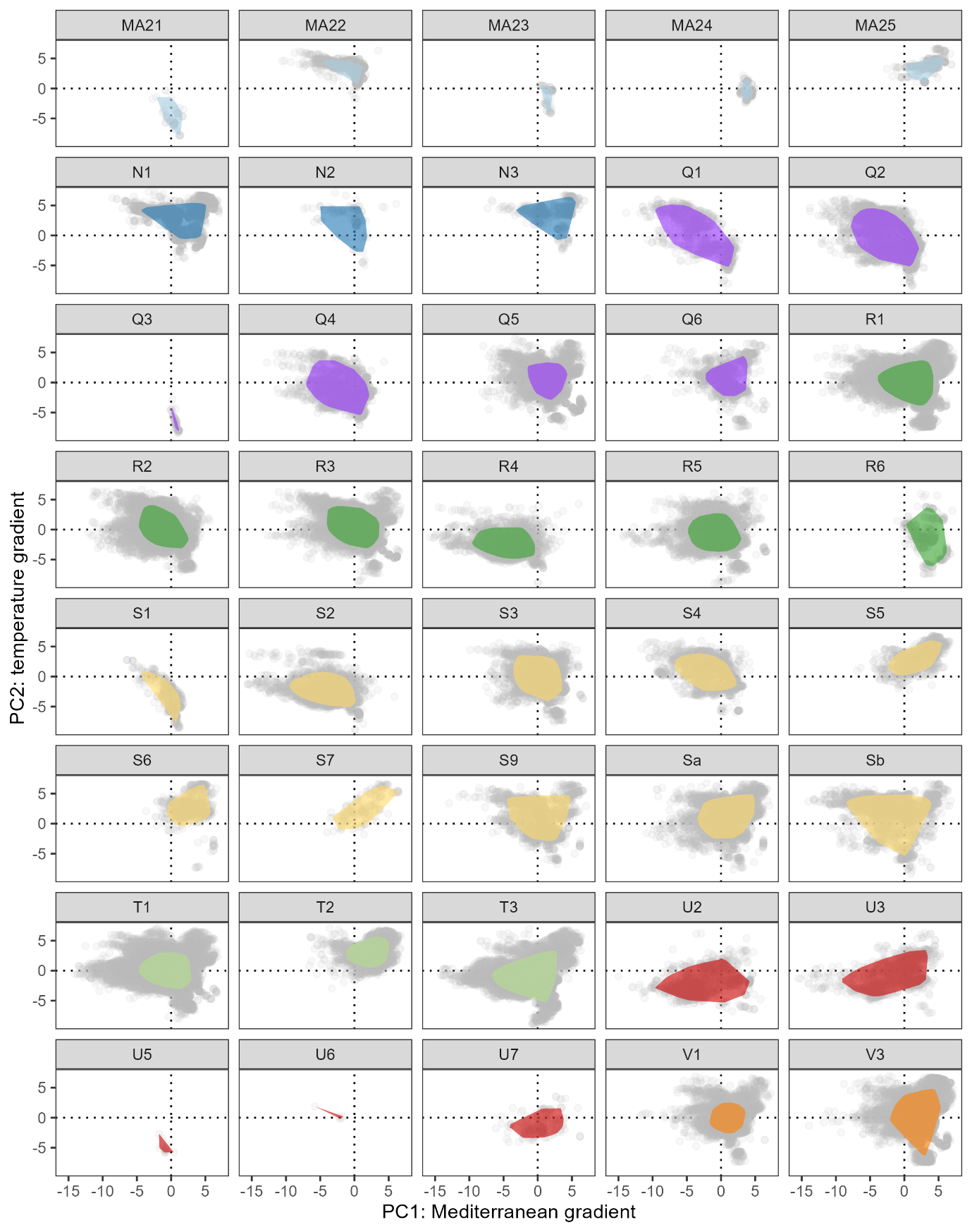


**Supplementary Fig. S5 | Distribution of the intermediate EUNIS level 2 habitats along the first and second principal components (PCs) of the 19 CHELSA bioclimatic variables**. The grey points show the position of the individual plots. The coloured polygons show the position of the minimum convex hulls that cover 80% of the plots. The colours refer to the different EUNIS level 1 habitats and the codes to intermediate EUNIS level 2 habitat types (as listed in Supplementary Data 2).


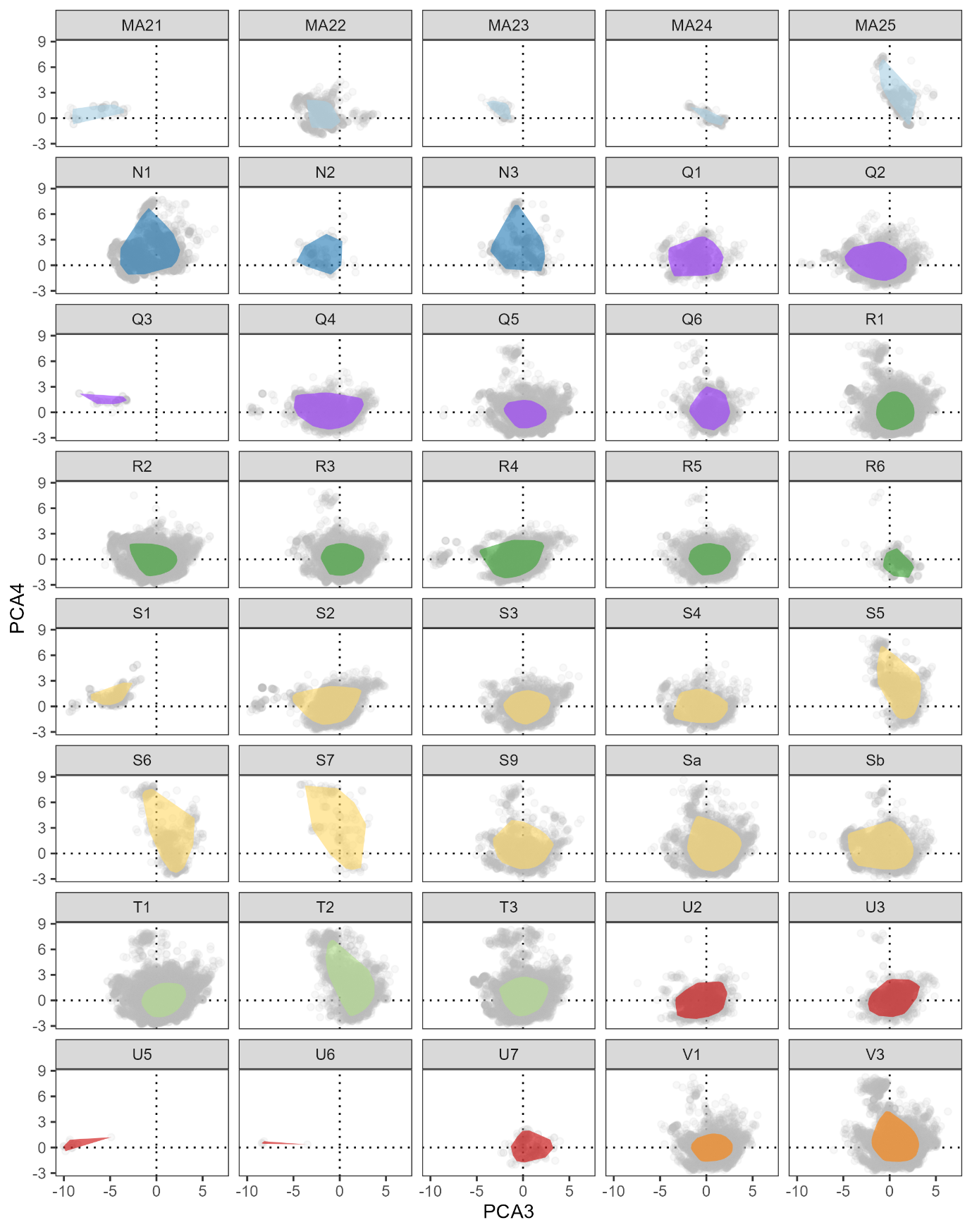


**Supplementary Fig. S6 | Distribution of the intermediate EUNIS level 2 habitats along the third and fourth principal components (PCs) of the 19 CHELSA bioclimatic variables**. The grey points show the position of the individual plots. The coloured polygons show the position of the minimum convex hulls that cover 80% of the plots. The colours refer to the different EUNIS level 1 habitats and the codes to intermediate EUNIS level 2 habitat types (as listed in Supplementary Data 2).


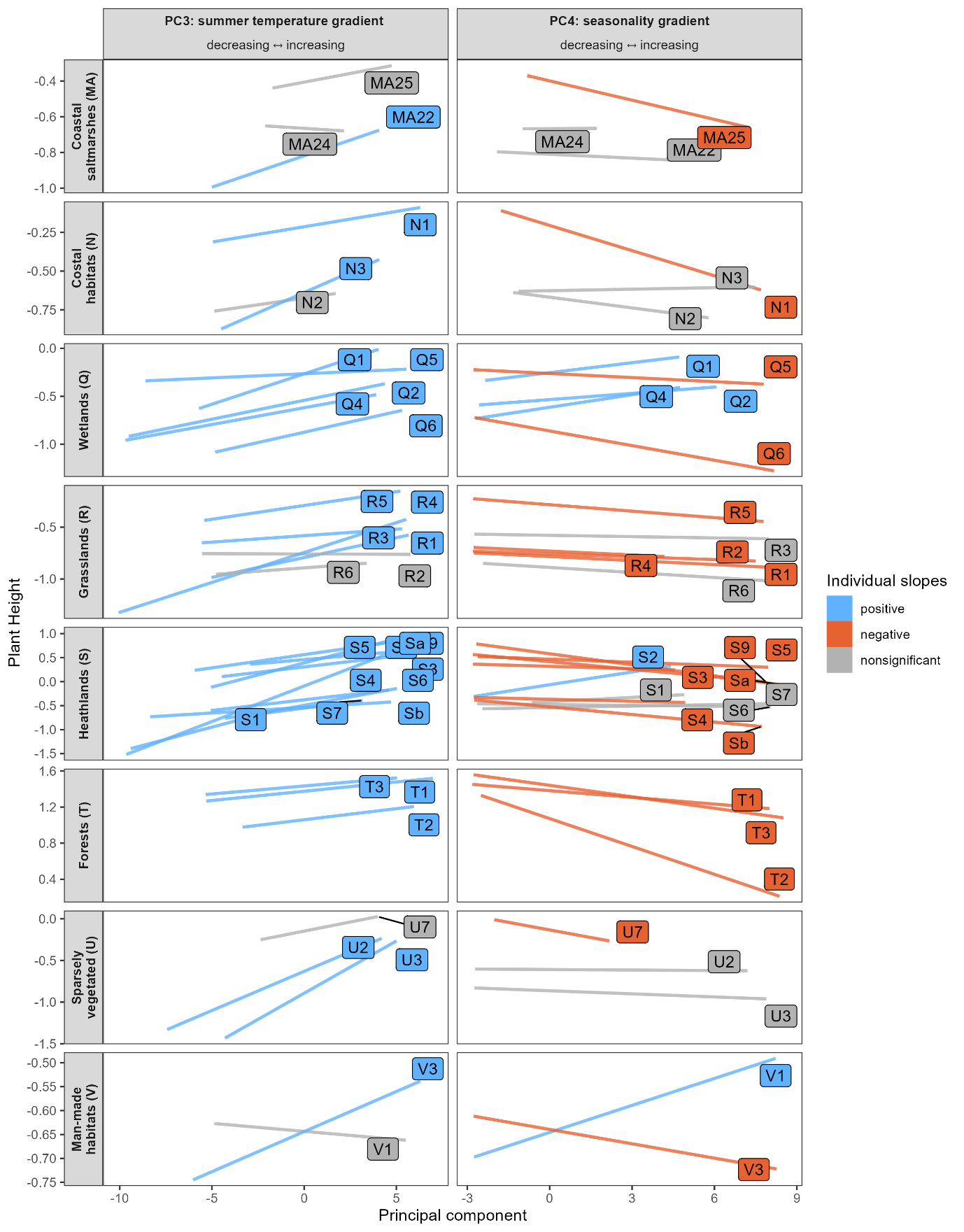


**Supplementary Fig. S7 | Effects of climate on plant height in intermediate-level habitats.** The graphs show the community-weighted means of plant height as a linear function of the third and fourth principal components (PCs) of the 19 CHELSA bioclimatic variables, obtained from generalized additive mixed-effects models. Significance was determined at p < 0.05 (based on separate two-sided t-tests). Slope estimates from habitats with fewer than 100 plot observations were omitted. Codes within boxes refer to habitat types (as listed in Supplementary Data 2).


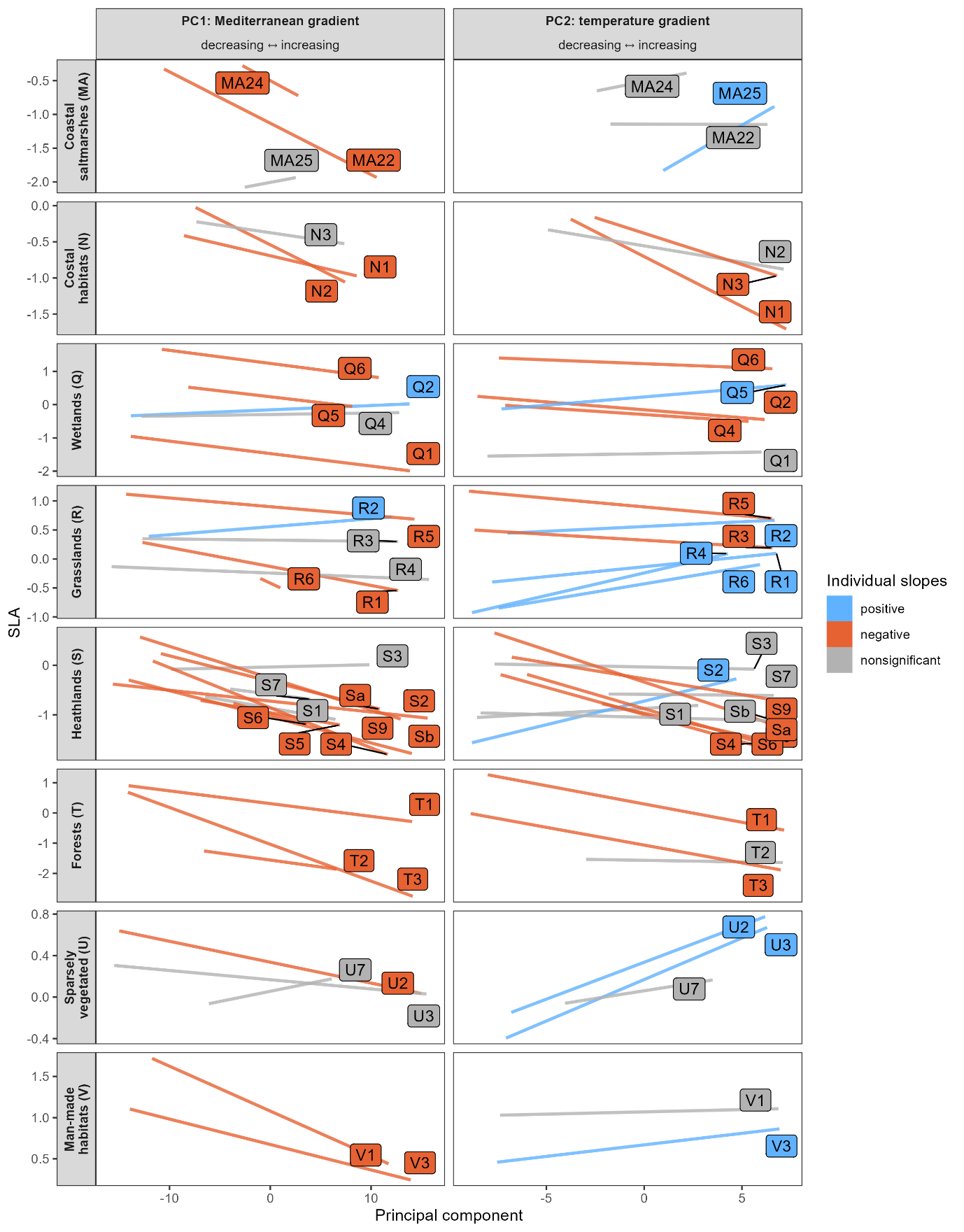


**Supplementary Fig. S8 | Effects of climate on specific leaf area (SLA) in intermediate habitats.** The graphs show the community-weighted means of plant SLA as a linear function of the first and second principal components (PCs) of the 19 CHELSA bioclimatic variables, obtained from generalized additive mixed-effects models. Significance was determined at p < 0.05 (based on separate two-sided t-tests). Slope estimates from habitats with fewer than 100 plot observations were omitted. Codes within boxes refer to habitat types (as listed in Supplementary Data 2).


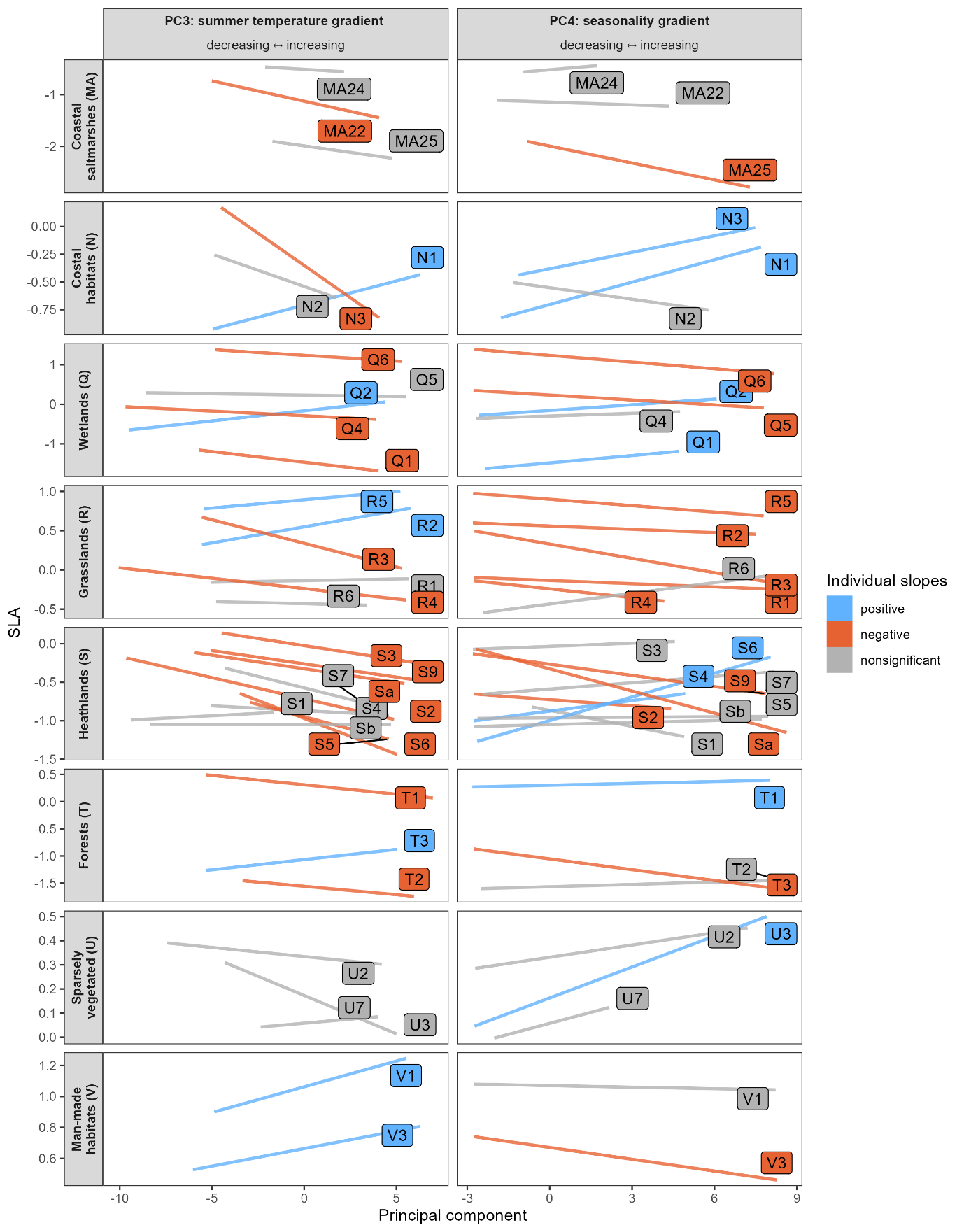


**Supplementary Fig. S9 | Effects of climate on specific leaf area (SLA) in intermediate habitats.** The graphs show the community-weighted means of plant SLA as a linear function of the third and fourth principal components (PCs) of the 19 CHELSA bioclimatic variables, obtained from generalized additive mixed-effects models. Significance was determined at p < 0.05 (based on separate two-sided t-tests). Slope estimates from habitats with fewer than 100 plot observations were omitted. Codes within boxes refer to habitat types (as listed in Supplementary Data 2).


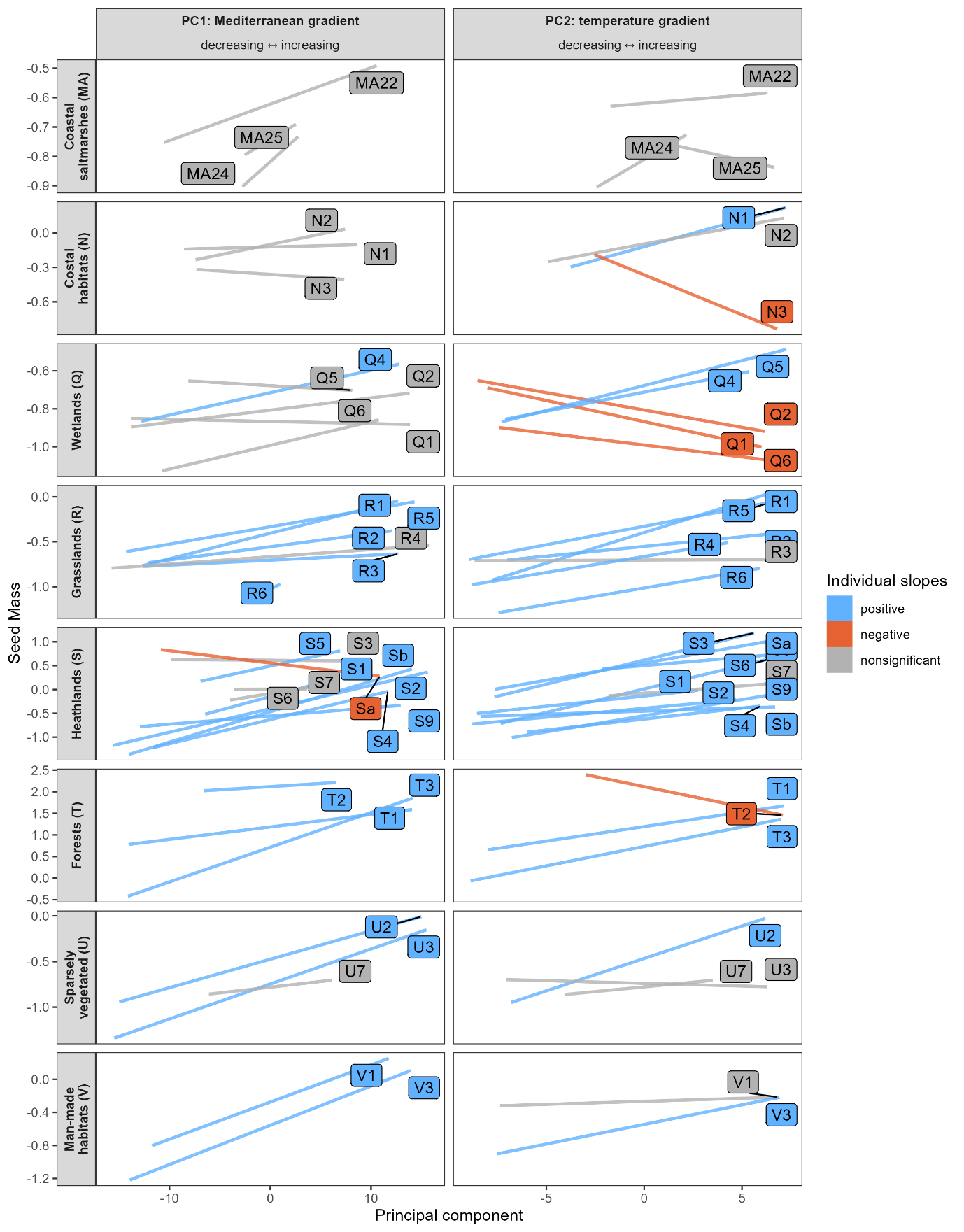


**Supplementary Fig. S10 | Effects of climate on seed mass in intermediate habitats.** The graphs show the community-weighted means of plant seed mass as a linear function of the first and second principal components (PCs) of the 19 CHELSA bioclimatic variables, obtained from generalized additive mixed-effects models. Significance was determined at p < 0.05 (based on separate two-sided t-tests). Slope estimates from habitats with fewer than 100 plot observations were omitted. Codes within boxes refer to habitat types (as listed in Supplementary Data 2).


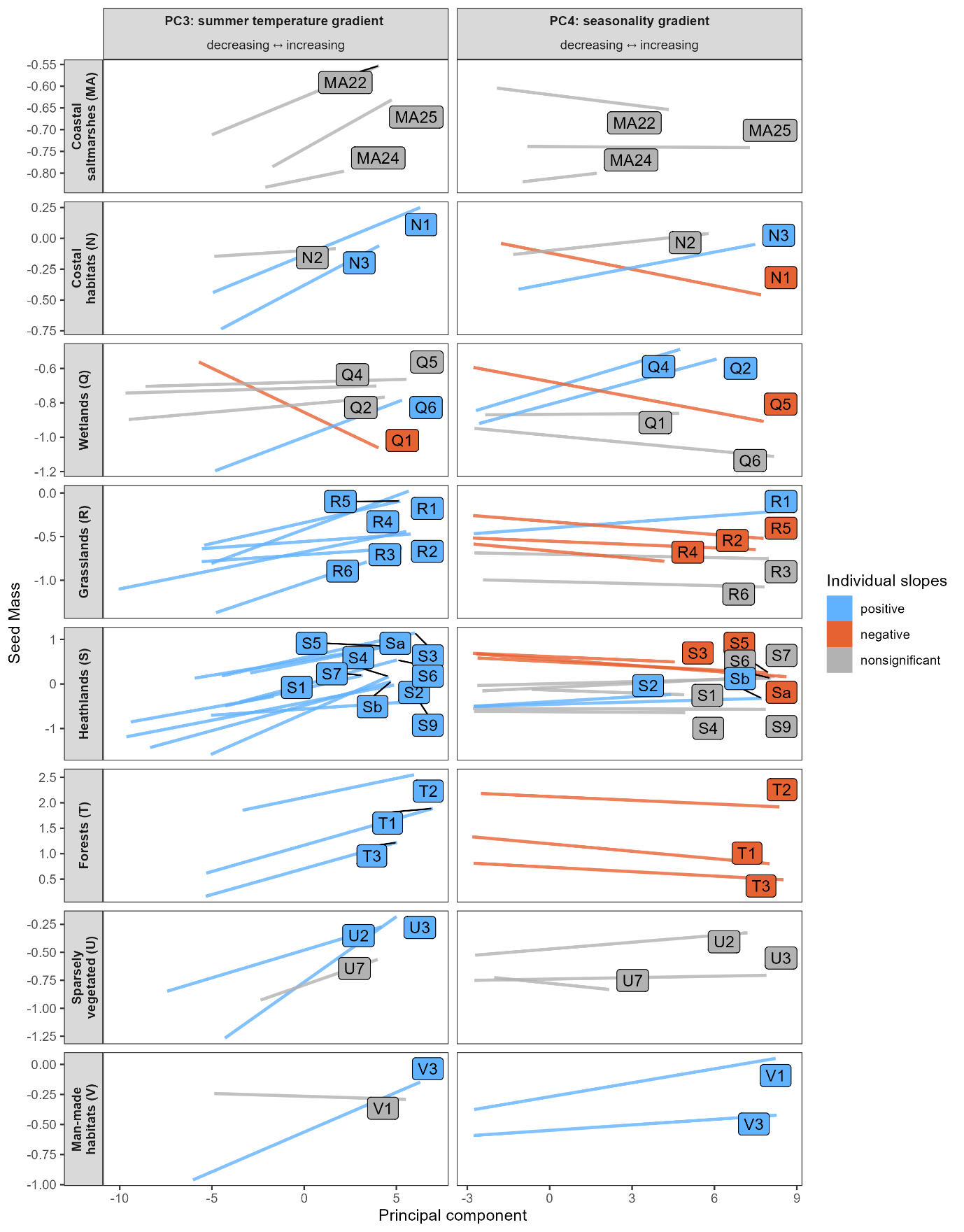


**Supplementary Fig. S11 | Effects of climate on seed mass in intermediate habitats.** The graphs show the community-weighted means of plant seed mass as a linear function of the third and fourth principal components (PCs) of the 19 CHELSA bioclimatic variables, obtained from generalized additive mixed-effects models. Significance was determined at p < 0.05 (based on separate two-sided t-tests). Slope estimates from habitats with fewer than 100 plot observations were omitted. Codes within boxes refer to habitat types (as listed in Supplementary Data 2).


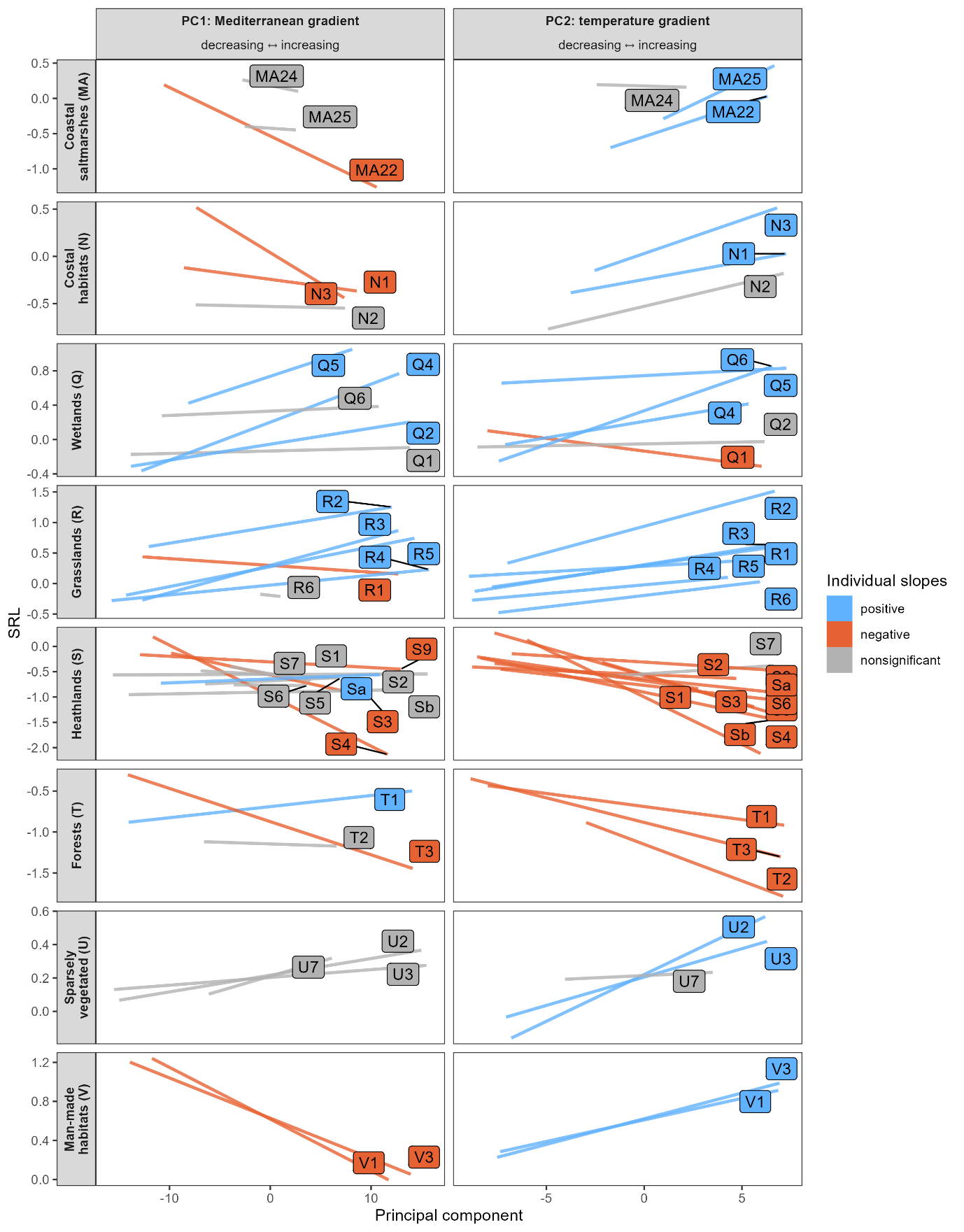


**Supplementary Fig. S12 | Effects of climate on specific root length (SRL) in intermediate habitats.** The graphs show the community-weighted means of plant SRL as a linear function of the first and second principal components (PCs) of the 19 CHELSA bioclimatic variables, obtained from generalized additive mixed-effects models. Significance was determined at p < 0.05 (based on separate two-sided t-tests). Slope estimates from habitats with fewer than 100 plot observations were omitted. Codes within boxes refer to habitat types (as listed in Supplementary Data 2).


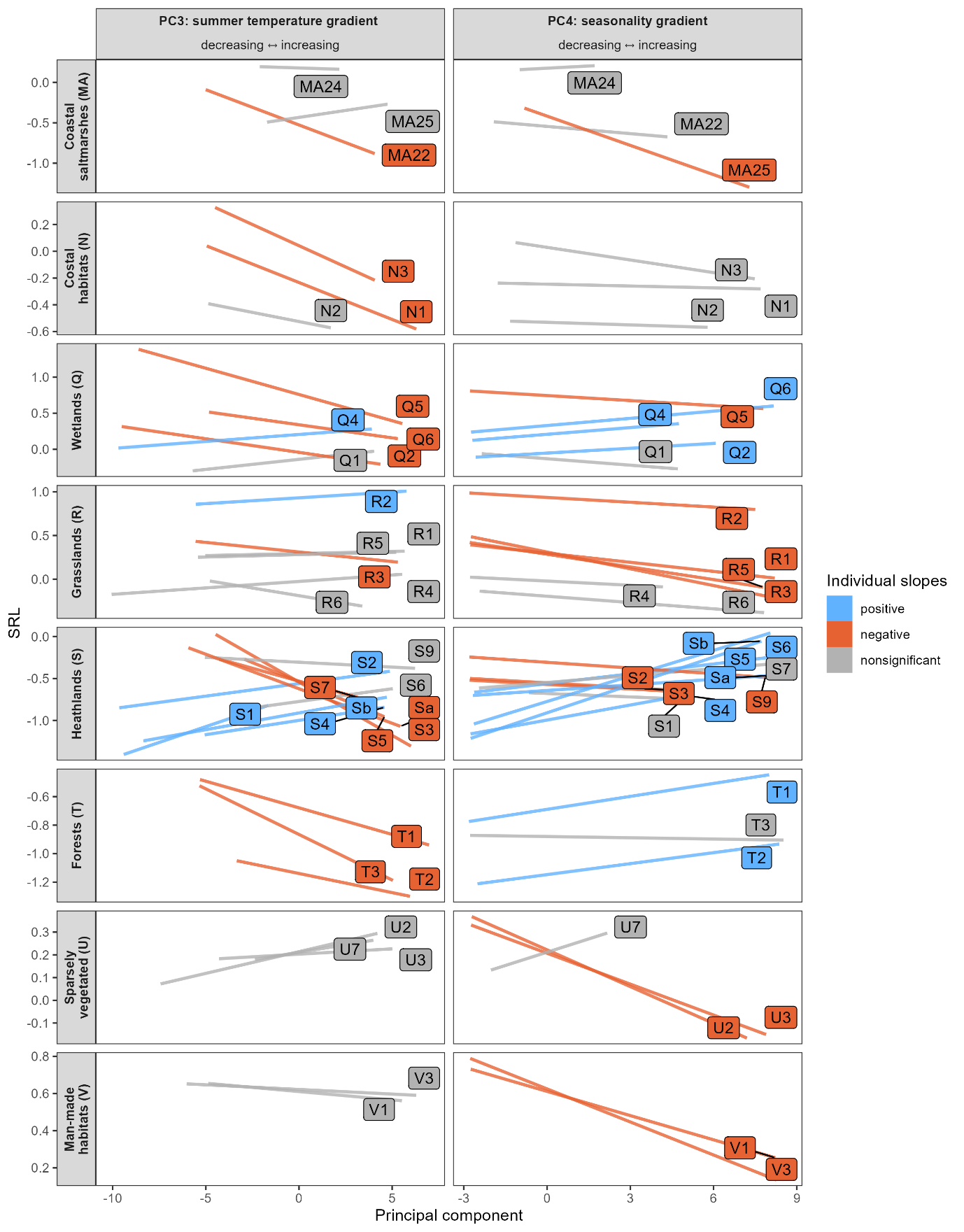


**Supplementary Fig. S13 | Effects of climate on specific root length (SRL) in intermediate habitats.** The graphs show the community-weighted means of plant SRL as a linear function of the third and fourth principal components (PCs) of the 19 CHELSA bioclimatic variables, obtained from generalized additive mixed-effects models. Significance was determined at p < 0.05 (based on separate two-sided t-tests). Slope estimates from habitats with fewer than 100 plot observations were omitted. Codes within boxes refer to habitat types (as listed in Supplementary Data 2).


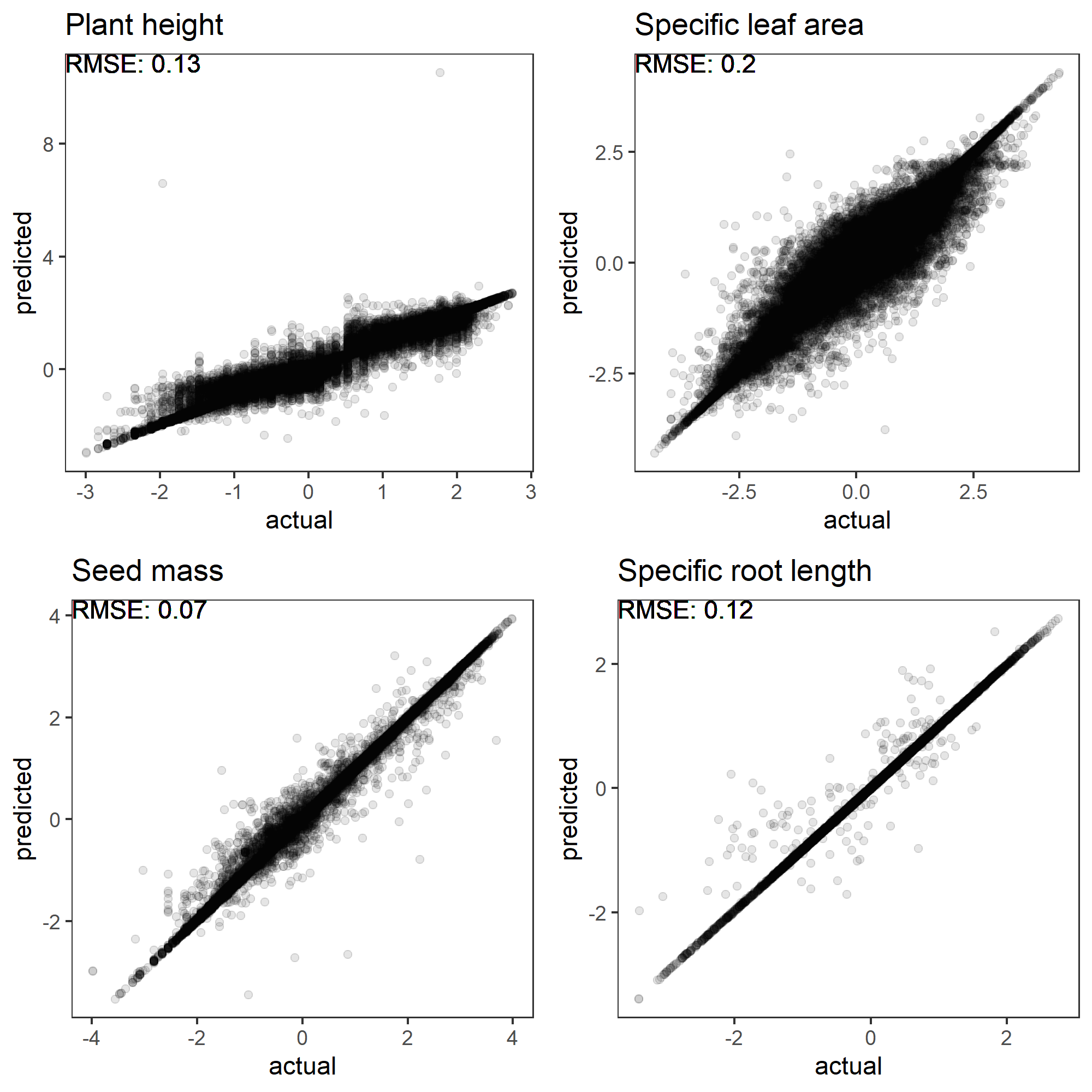


**Supplementary Fig. S14 | Available versus predicted species-level z-transformed values for four plant traits from the gap-filled dataset of the TRY plant database^30^.** Predictions were conducted with Bayesian Hierarchical Probabilistic Matrix Factorization^55^, based on 33 traits and the taxonomy from the TRY plant trait database^30^. RMSE = root-mean-square error.


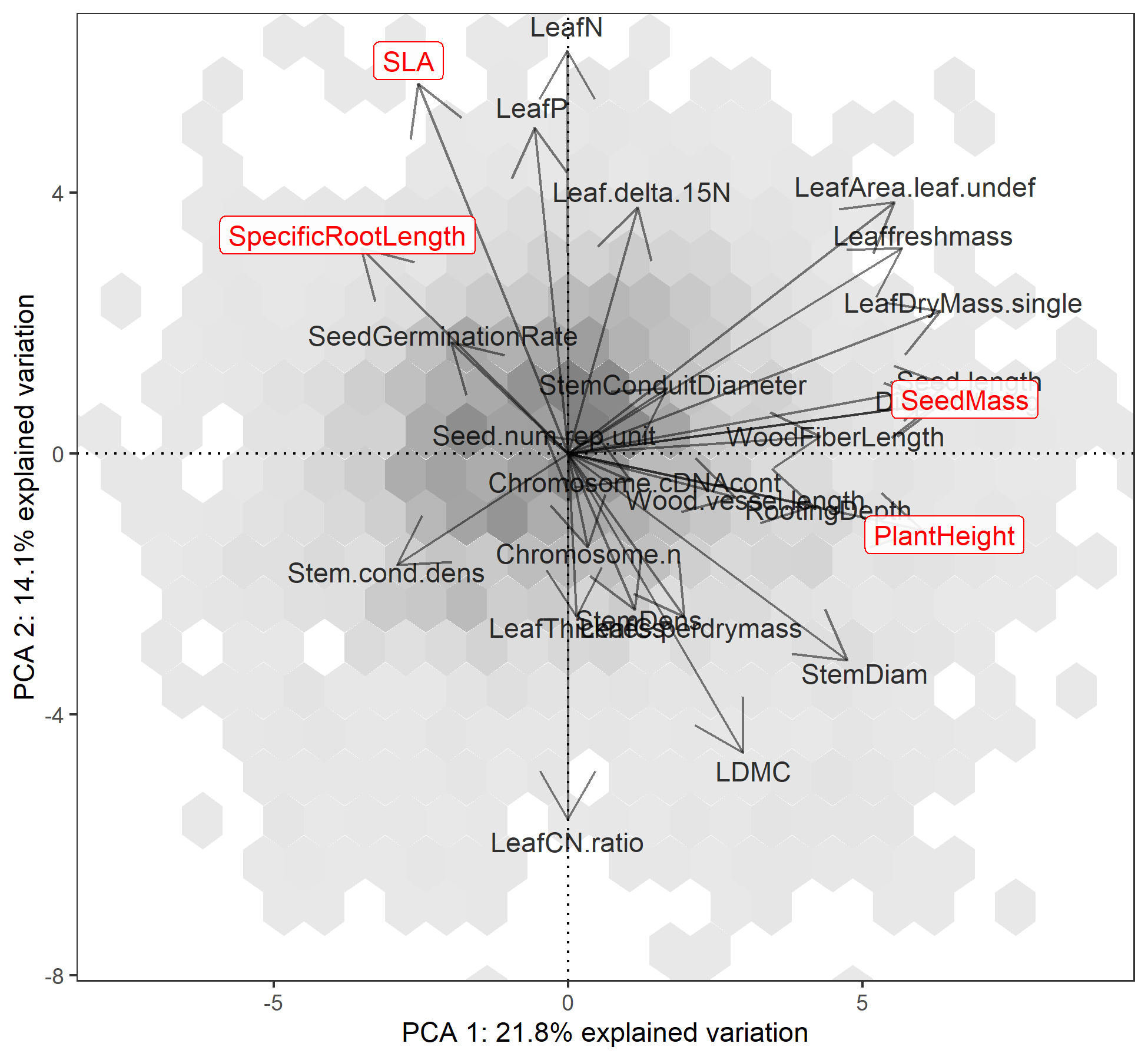


Chromosome.cDNAcont - chromosome cDNA content, Chromosome.n - chromosome cDNA content, Disp.unit.leng - Dispersal unit length, LDMC - leaf dry matter content, Leaf.delta.15N - Leaf nitrogen isotope signature, LeafArea.leaf.undef - Leaf area (in case of compound leaves: leaf), LeafC.perdrymass - Leaf carbon content per leaf dry mass, LeafCN.ratio - Leaf carbon/nitrogen ratio, LeafDryMass.single - Leaf dry mass, Leaffreshmass - Leaf fresh mass, LeafN - Leaf nitrogen content per leaf dry mass, LeafP - Leaf phosphorus content per leaf dry mass, LeafThickness - Leaf thickness, LeafWaterCont - Leaf water content per leaf dry mass, LeafWidth - Leaf width, PlantHeight - Plant height, RootingDepth - Root rooting depth, Seed.length - Seed length, Seed.num.rep.unit - Seed number per reproductive unit, SeedGerminationRate - Seed germination rate, SeedMass - Seed dry mass, SLA - specific leaf area, SpecificRootLength - specific root length, Stem.cond.dens - Stem conduit density (vessels and tracheids), StemConduitDiameter - Stem conduit diameter (vessels, tracheids), StemDens - Stem specific density or wood density, StemDiam - Stem diameter, Wood.vessel.length - Wood vessel element length, WoodFiberLength - Wood fibre lengths.

**Supplementary Fig. S15 | Relationships between the 28 species-level or genus-level traits for 6,715 species along the first and second principal component axes (PCA)** in the gap-filled dataset of the TRY plant trait database^30^. All traits were log-transformed and normalized prior to the analysis.


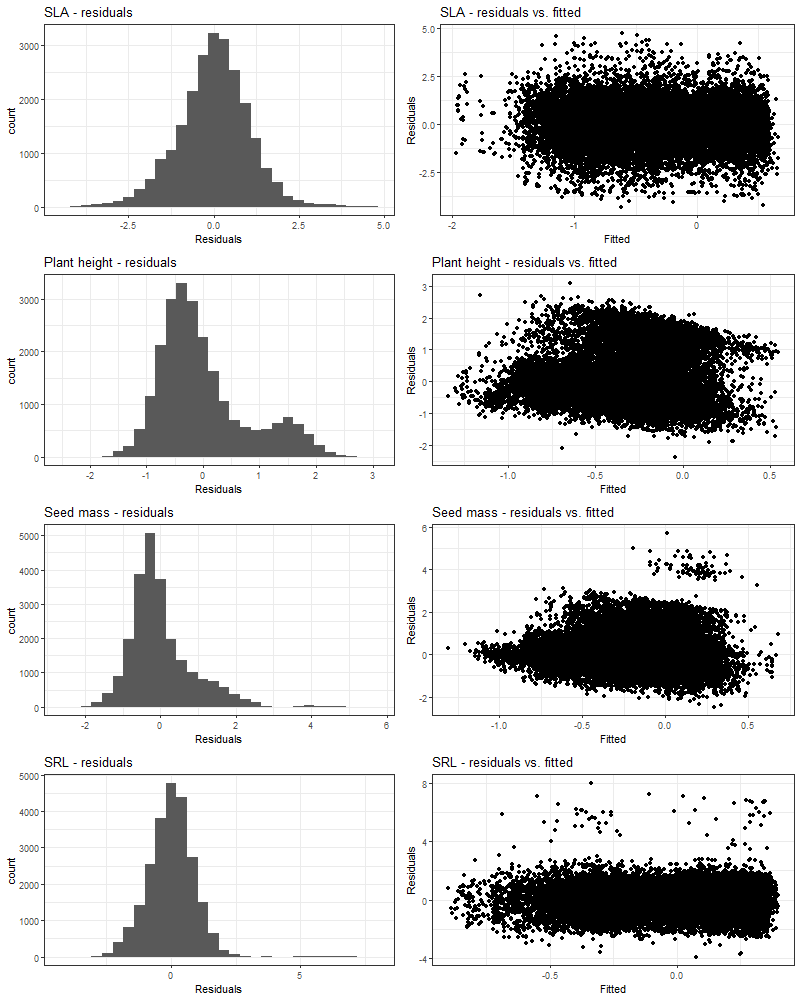


**Supplementary Fig. S16 | Residual plots for the generalized additive mixed-effects model number one.** Each row shows the residuals from a separate model for the four different trait CWMs. Left panels show the histograms of the model residuals which tend to follow a normal distribution. Right panels show the relationship between the predicted values for the CWMs and the respective residuals. SLA: specific leaf area, SRL: specific root length.


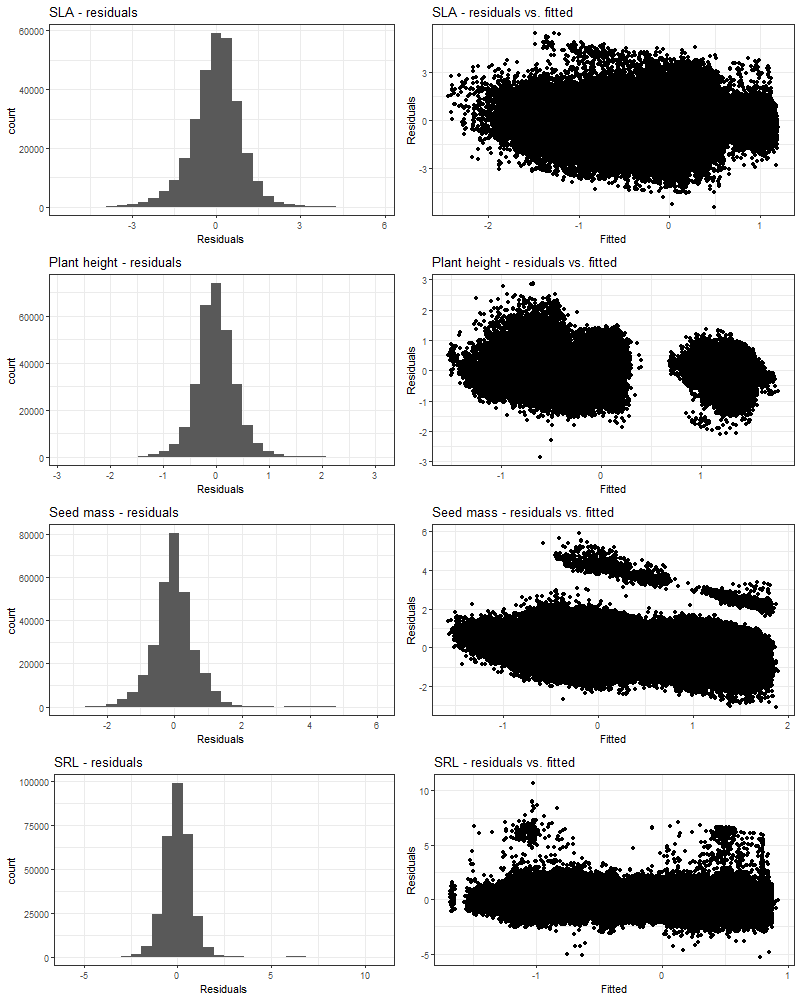


**Supplementary Fig. S17 | Residual plots for the generalized additive mixed-effects model number two.** Each row shows the residuals from a separate model for the four different trait CWMs. Left panels show the histograms of the model residuals which tend to follow a normal distribution. Right panels show the relationship between the predicted values for the CWMs and the respective residuals. SLA: specific leaf area, SRL: specific root length.


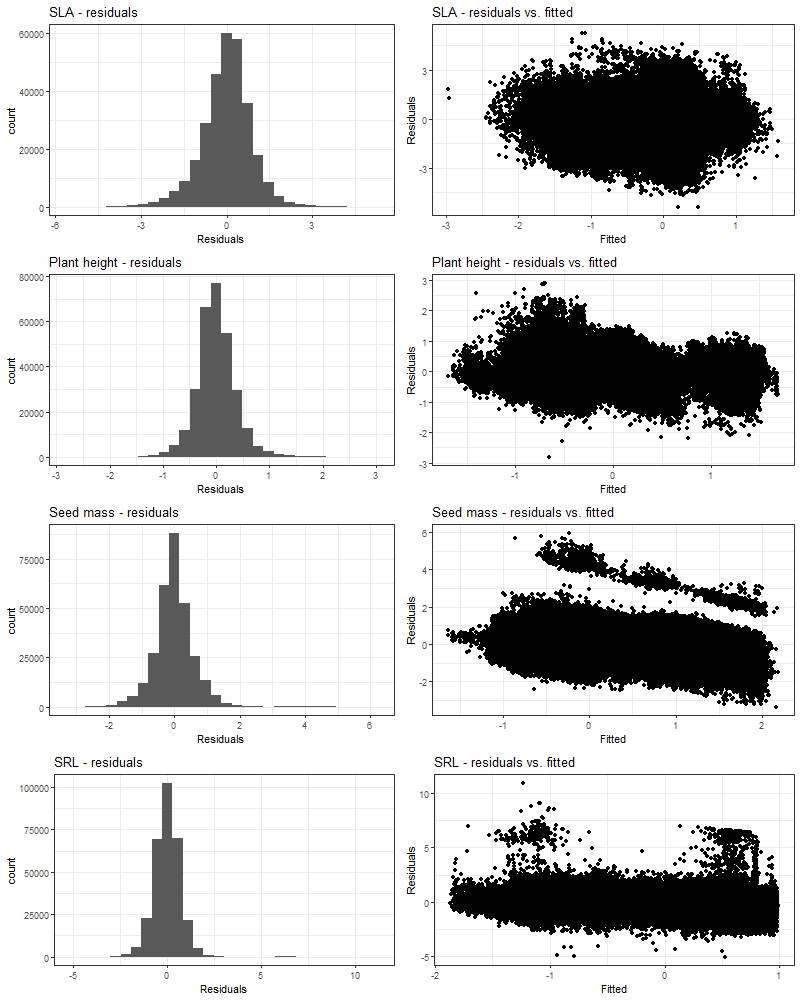


**Supplementary Fig. S18 | Residual plots for the generalized additive mixed-effects model number three.** Each row shows the residuals from a separate model for the four different trait CWMs. Left panels show the histograms of the model residuals which tend to follow a normal distribution. Right panels show the relationship between the predicted values for the CWMs and the respective residuals. SLA: specific leaf area, SRL: specific root length.


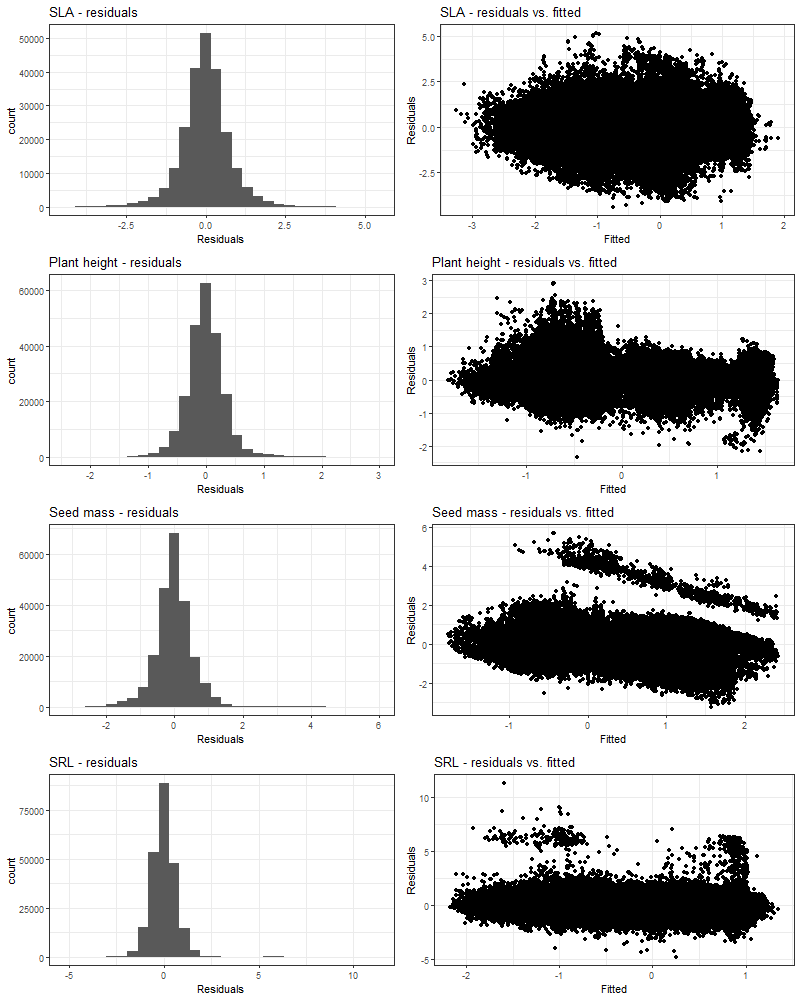


**Supplementary Fig. S19 | Residual plots for the generalized additive mixed-effects model number four.** Each row shows the residuals from a separate model for the four different trait CWMs. Left panels show the histograms of the model residuals which tend to follow a normal distribution. Right panels show the relationship between the predicted values for the CWMs and the respective residuals. SLA: specific leaf area, SRL: specific root length.


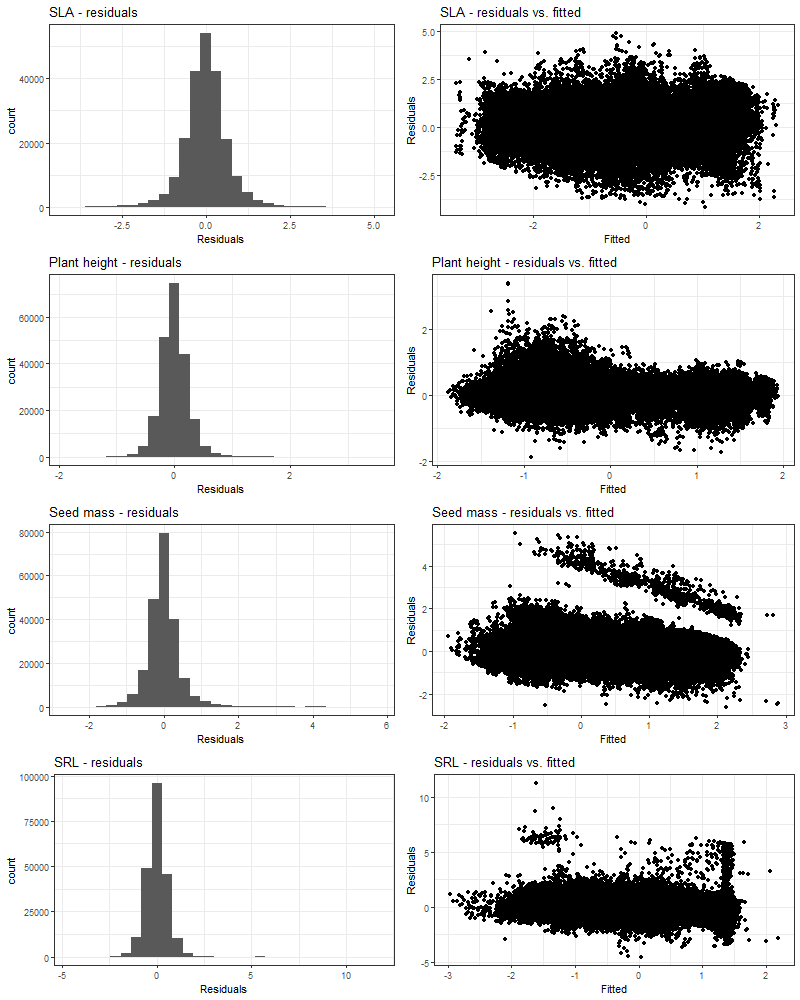


**Supplementary Fig. S20 | Residual plots for the generalized additive mixed-effects model number five.** Each row shows the residuals from a separate model for the four different trait CWMs. Left panels show the histograms of the model residuals which tend to follow a normal distribution. Right panels show the relationship between the predicted values for the CWMs and the respective residuals. SLA: specific leaf area, SRL: specific root length.


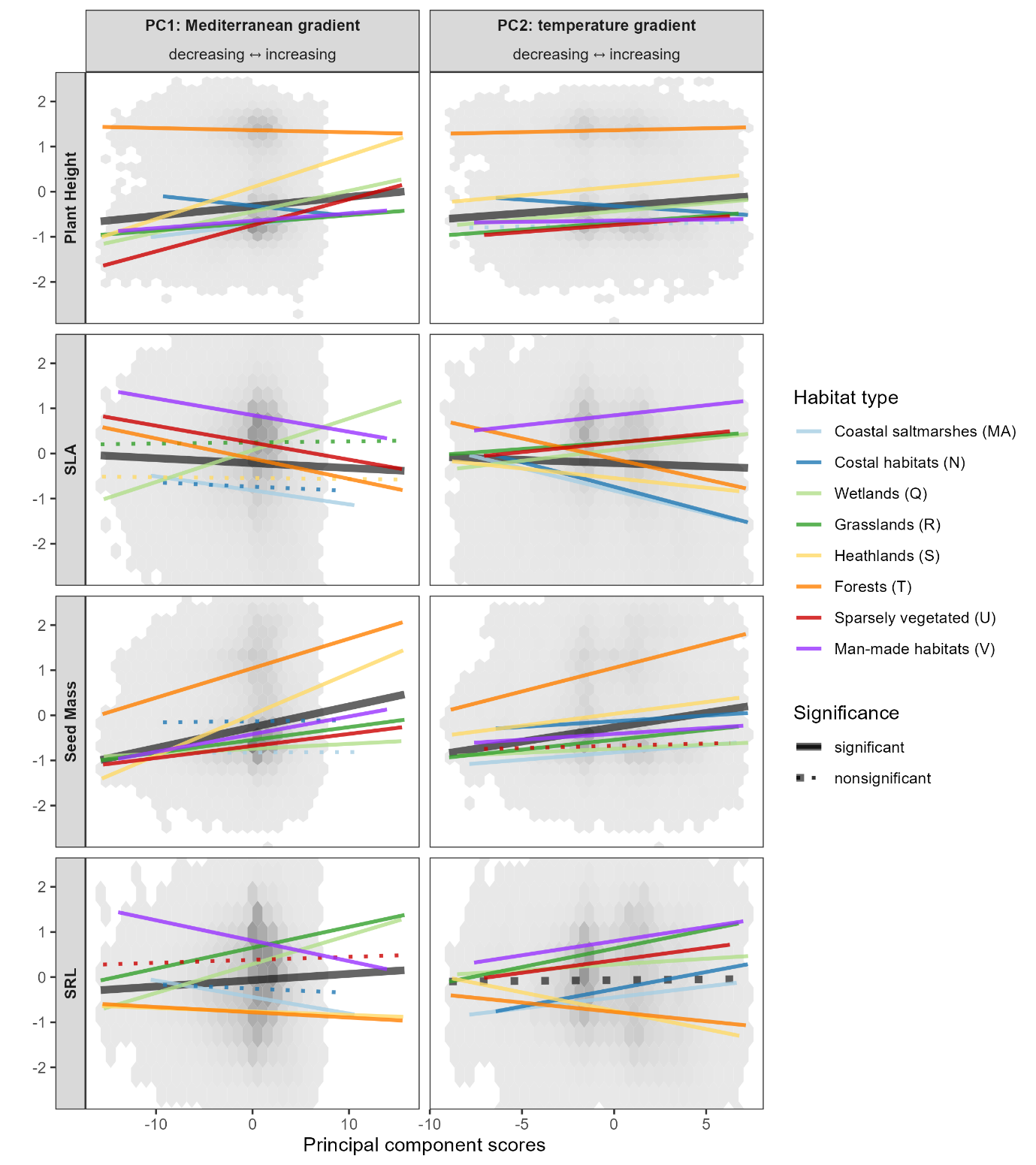


**Supplementary Fig. S21 | Effects of climate on four plant traits in broad habitats.** The graphs show the community-weighted means of four plant functional traits as linear functions of the first and second principal components (PCs) of the 19 CHELSA bioclimatic variables, obtained with **weighted** generalized additive mixed-effects models. Slopes show the relationships across all (black) and within the most broadly defined habitats of the EUNIS classification (colours). Solid lines indicate significant relationships at p < 0.05 (based on separate two-sided t-tests). Grey hexagons show the distribution of plot-level observations. SLA: specific leaf area, SRL: specific root length.


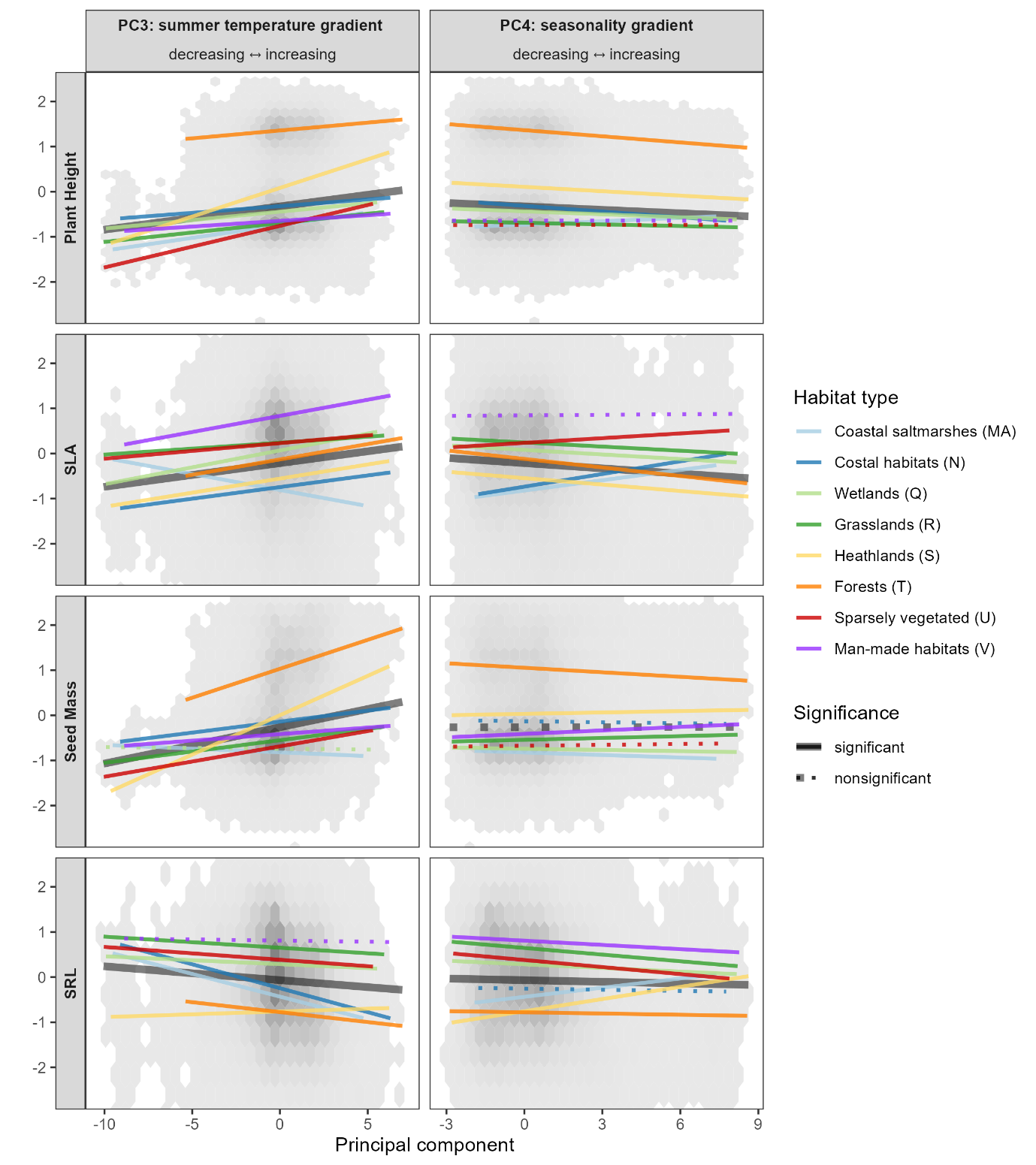


**Supplementary Fig. S22 | Effects of climate on four plant traits in broad habitats.** The graphs show the community-weighted means of four plant functional traits as linear functions of the third and fourth principal components (PCs) of the 19 CHELSA bioclimatic variables, obtained with **weighted** generalized additive mixed-effects models. Slopes show the relationships across all (black) and within the most broadly defined habitats of the EUNIS classification (colours). Solid lines indicate significant relationships at p < 0.05 (based on separate two-sided t-tests). Grey hexagons show the distribution of plot-level observations. SLA: specific leaf area, SRL: specific root length.


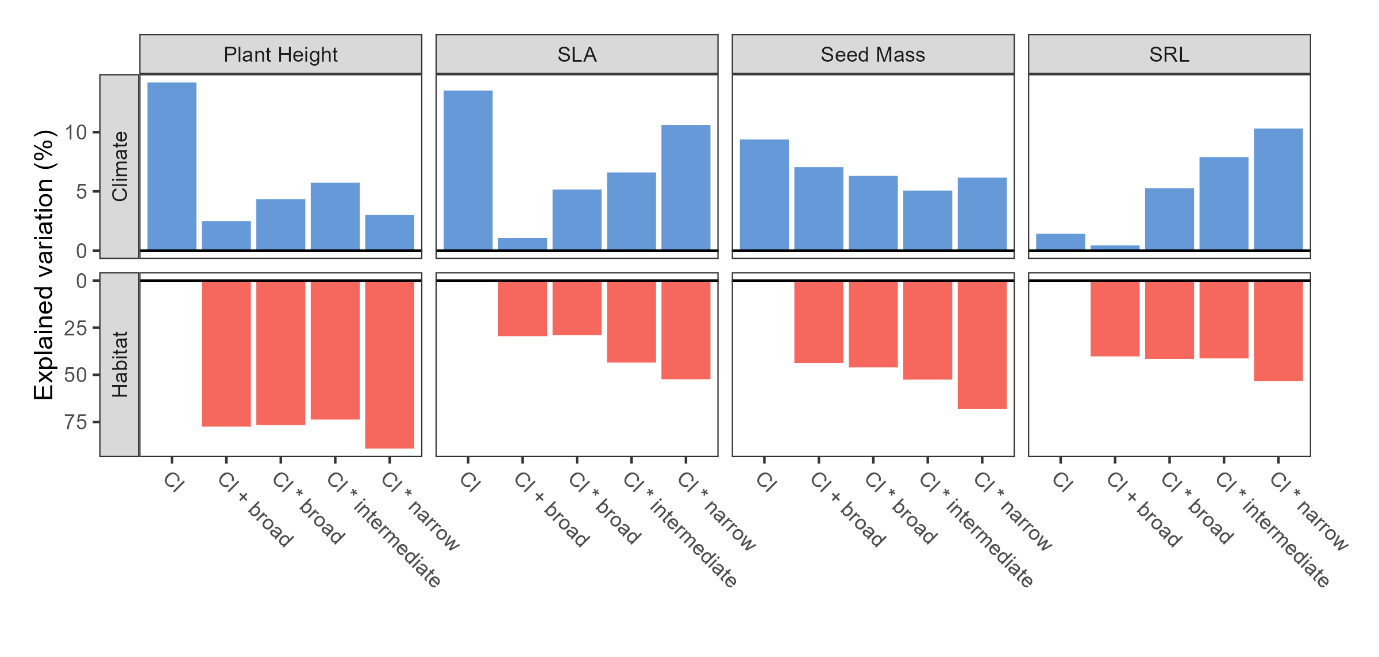


**Supplementary Fig. S23 | Proportion of variation in plant traits explained by climate and increasingly narrow habitat definitions**. The graphs show the marginal R² values from **weighted** generalized additive mixed-effects models for the linear dependence of the community-weighted means of four plant functional traits on habitat (red bars) and the general and habitat-specific effects of climate (blue bars). Model complexity increases from left to right. CL: effects of climate across all plots or habitats, modelled with fixed effects of the four principal components of the 19 bioclimatic variables. CL + broad: fixed effects of climate plus random intercept effects of broad habitats. Cl * broad/intermediate/narrow: fixed effects of climate plus the random effects of broad, intermediate, or narrowly defined habitats plus the habitat-specific effects of climate, modelled with random-slope effects between the climatic principal components and the respective habitats. SLA: specific leaf area, SRL: specific root length.


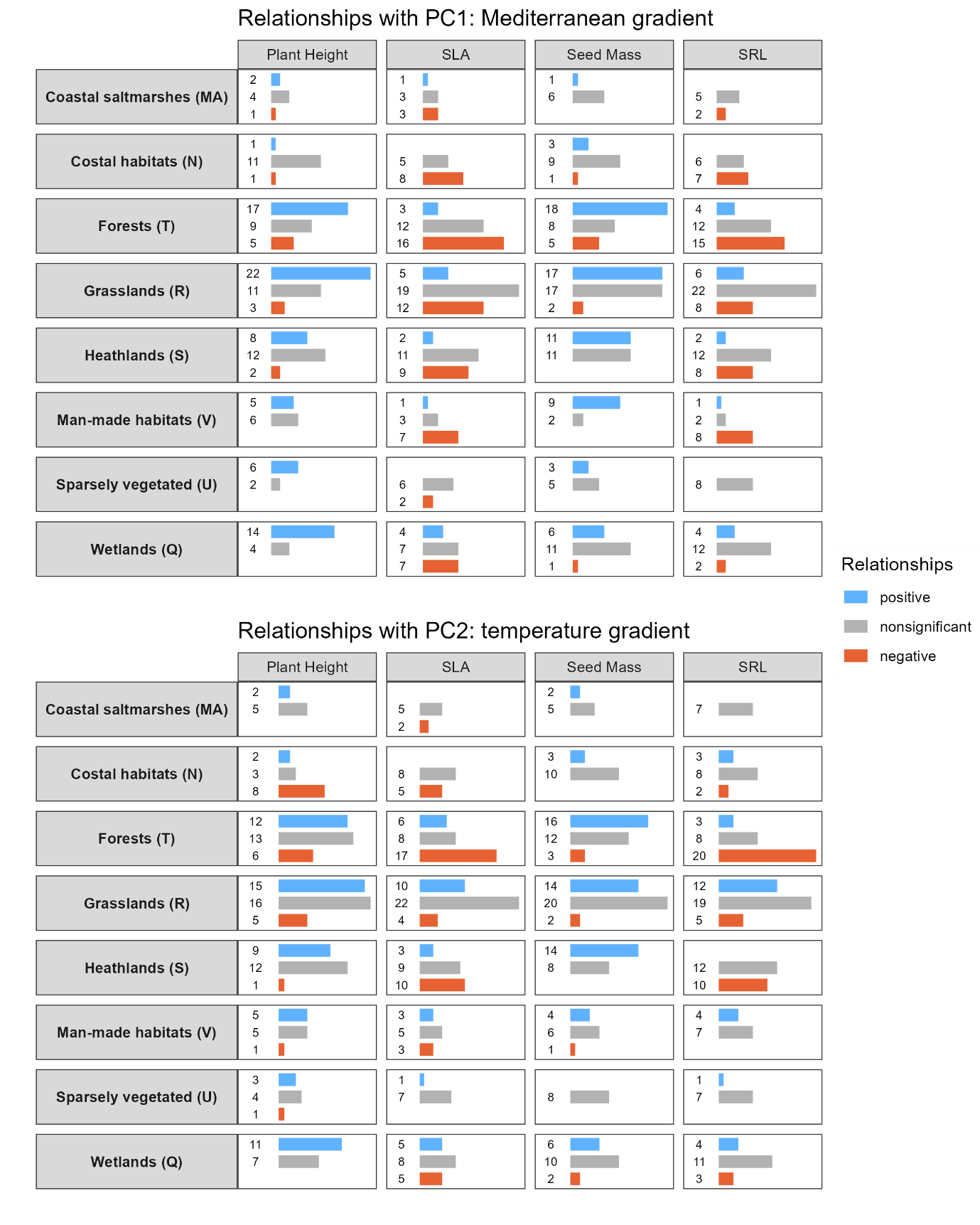


**Supplementary Fig. S24 | Effects of climate on plant traits in the most narrowly defined habitats.** The graphs show the number of slope estimates between the first and second principal components (PCs) of the 19 CHELSA bioclimatic variables and the community-weighted mean of four functional traits, calculated with **weighted** generalized additive mixed-effects models. Colouring indicates the expression and significance of the relationship. Significance was determined at p < 0.05 (based on separate two-sided t-tests). Slope estimates from habitats with fewer than 100 plot observations were omitted. SLA: specific leaf area, SRL: specific root length.


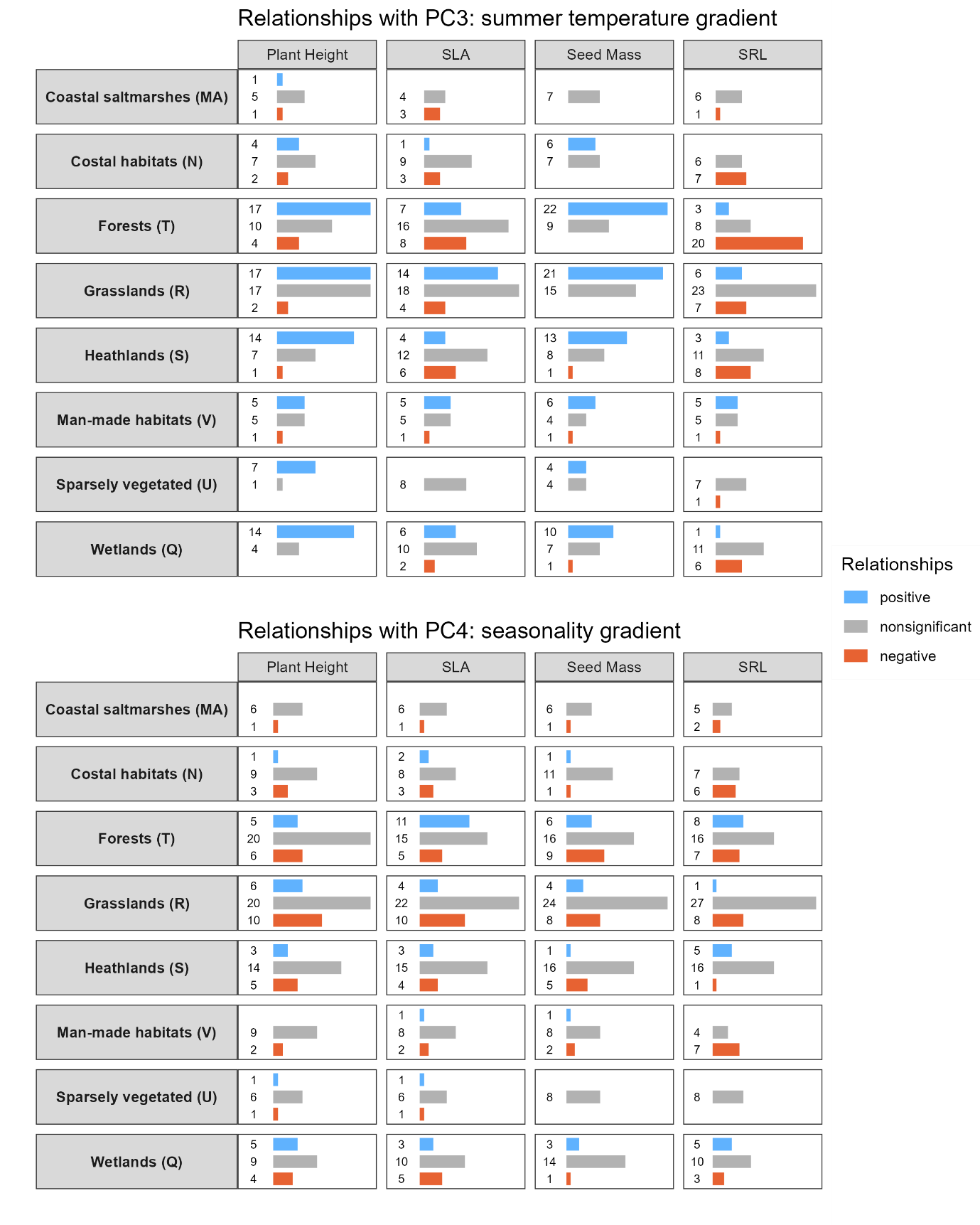


**Supplementary Fig. S25 | Effects of climate on plant traits in the most narrowly defined habitats.** The graphs show the number of slope estimates between the third and fourth principal components (PCs) of the 19 CHELSA bioclimatic variables and the community-weighted mean of four functional traits, calculated with **weighted** generalized additive mixed-effects models. Colouring indicates the expression and significance of the relationship. Significance was determined at p < 0.05 (based on separate two-sided t-tests). Slope estimates from habitats with fewer than 100 plot observations were omitted. SLA: specific leaf area, SRL: specific root length.
