# Supplementary material for: Climate-trait relationships exhibit strong habitat specificity in plant communities across Europe
Source: Nat Commun. 2023 Feb 9;14:712. doi: 10.1038/s41467-023-36240-6 (PMC9911725; doi:10.1038/s41467-023-36240-6)
Supplement: Supplementary file 3 — Description of Additional Supplementary Information [file 41467_2023_36240_MOESM3_ESM.pdf]

Description of Additional Supplementary Information

File name: Supplementary Data 1

Description: Databases used for this study together with their GIVD – Global Index of Vegetation-Plot Databases (www.givd.info)

File name: Supplementary Data 2

Description: Number of plots in intermediate and most narrowly defined habitats, following the EUNIS habitat classification. Habitats with NA include plots that could not be assigned unequivocally.
